# Region-wise landmarks-based feature extraction employing SIFT, SURF, and ORB feature descriptors to recognize Monozygotic twins from 2D/3D Facial Images

**DOI:** 10.12688/f1000research.162911.2

**Published:** 2025-06-20

**Authors:** Gangothri Sanil, Krishna Prakasha K, Srikanth Prabhu, Vinod Nayak, Aparna Jayakala

**Affiliations:** 1 Information Communication Technology, Manipal Institute of Technology (MIT), Manipal Academy of Higher Education, Manipal, 576104, India; 2 Information Communication Technology, Manipal Academy of Higher Education (MAHE),, Manipal Academy of Higher Education, Manipal Institute of Technology (MIT), Manipal, 576104, India; 3 Computer science and Engineering,, Manipal Academy of Higher Education, Manipal Institute of Technology (MIT), Manipal, India, 576104, India; 4 Forensic Medicine and Toxicology, Kasturba Medical College(KMC), Manipal Academy of Higher Education, Manipal, India, 576104, India

**Keywords:** Facial images; 468 Landmarks; Local features; Key points; Feature Descriptor; Monozy-gotic twins; Machine Learning

## Abstract

**Background:**

In computer vision and image processing, face recognition is increasingly popular field of research that identifies similar faces in a picture and assigns a suitable label. It is one of the desired detection techniques employed in forensics for criminal identification.

**Methods:**

This study explores face recognition system for monozygotic twins utilizing three widely recognized feature descriptor algorithms: Scale-Invariant Feature Transform (SIFT), Speeded-Up Robust Features (SURF), and Oriented Fast and Rotated BRIEF (ORB)—with region-specific facial landmarks. These landmarks were extracted from 468 points detected through the MediaPipe framework, which enables simultaneous recognition of multiple faces. Quantitative similarity metrics t served as inputs for four classification methods: Support Vector Machine (SVM), eXtreme Gradient Boost (XGBoost), Light Gradient Boost Machine (LGBM), and Nearest Centroid (NC). The effectiveness of these algorithms was tested and validated using challenging ND Twins and 3D TEC datasets, the most difficult data sets for 2D and 3D face recognition research at Notre Dame University.

**Results:**

Testing with Notre Dame University’s challenging ND Twins and 3D TEC datasets revealed significant performance differences. Results demonstrated that 2D facial images achieved notably higher recognition accuracy than 3D images. The 2D images produced accuracy of 88% (SVM), 83% (LGBM), 83% (XGBoost), and 79% (NC). In contrast, the 3D TEC dataset yielded a lower accuracy r of 74%, 72%, 72%, and 70%, with the same classifiers.

**Conclusion:**

The hybrid feature extraction approach proved most effective, with maximum accuracy rates reaching 88% for 2D facial images and 74% for 3D facial images. This work contributes significantly to forensic science by enhancing the reliability of facial recognition systems when confronted with indistinguishable facial characteristics of monozygotic twins.

**Abbreviations**
3D TEC: 3D Twins Expression Challenge
CM: Confusion matrix
DLIB: Digital Library
DNA: Deoxyribonucleic Acid
FAR: False Accept Rate
FRR: False Reject Rate
FRS: Face Recognition System
LGBM: Light Gradient Boosting Machine
ND-Twins
: Notre Dame-Twins
NC: Nearest Centroid
ORB: Oriented Fast and Rotated BRIEF
RF: Random Forest
ROC: Receiver Operating Characteristics
SIFT: Scale-Invariant Feature Transform
SURF: Speeded Up Robust Feature
SVM: Support Vector Machine
TAR: True Accept Rate
TRR: True Reject Rate
XGBoost: Xtreme Gradient Boosting

## 1. Introduction

Face recognition, taken more broadly, refers to methods of recognizing or authenticating a person based on a digital representation of their face. Because face biometrics are non-intrusive and imaging equipment for people is widely available, the utilization of face recognition systems has improved significantly in recent years. ^1^ Face position, age, gender, lighting, and other changing conditions are some difficulties in face identification. Identifying monozygotic twins, or identical twins, is one of the main problems in this field. ^2^ Numerous industries, including forensics, healthcare, and even targeted marketing, are significantly affected by the challenging task of recognizing identical twins. Identical twins have similar faces and facial features, causing a reduction in face recognition accuracy. Identical twins and lookalikes show the highest degree of visual similarity, making them the most challenging situations for facial recognition algorithms. ^3^ In the case of identical twins that are genetically similar, it is believed to be extremely difficult to distinguish them using routine forensic DNA (Deoxyribonucleic Acid) testing. Existing twin identification technologies do not appear to be effective for identical twins. As a result, the current effort is based on a binary categorization known as identical twin recognition. The study focuses on proposing a face recognition system to identify and authenticate identity twins in forensic-related crimes. Appearance-based and feature-based methods are the two primary categories of face-recognition algorithms. The authors have provided the feature-based technique for 2D face images in this paper considering various feature description algorithms. ^4^ By offering the following suggested solution, the proposed approach attempts to overcome the difficulties associated with twin identification. Three widely used feature descriptor techniques are SIFT, ORB, and SURF. These local feature descriptors have proven especially effective in twin recognition. ^5^ Several features were suggested and retrieved based on the salient points of the SIFT, SURF, ORB algorithms, and facial landmarks. The study finds facial differences more accurate for the recognition of identical twins by using the extracted features using three descriptors to determine them separately and using various combinations of these three approaches.

### 1.1 Motivation

**Figure 1.**
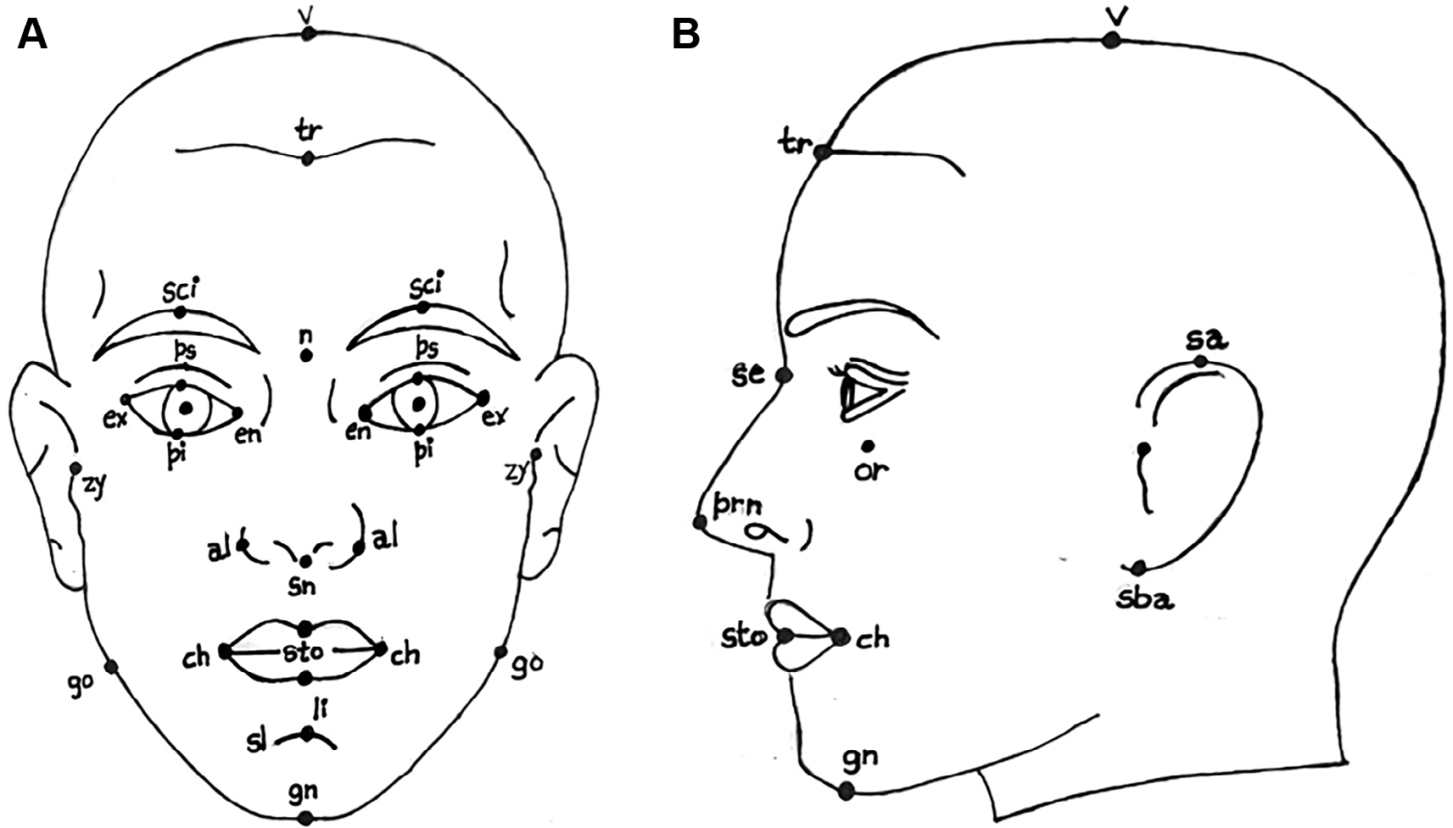
Identical twin faces. ^6^

Monozygotic and dizygotic twins are the two basic types of twins that exist. Monozygotic twins are the ones that result from the fertilization of a single egg that splits into two and are called identical twins. Dizygotic twins are the ones that result from the fertilization of two separate eggs and are called Fraternal Twins. The identical twin faces are depicted in Figure 1.

The existing automated twin recognition system does not work well for monozygotic twins. Finding differences between identical twins can be difficult due to their similar features, such as color, ears, noses, DNA, eyes, and fingerprints. In recent years, the birth rate of twins has increased along with the increase in crime. ^7^ The similarities between identical twins have been taken advantage of and used for fraud and criminal activities, hence, there is an immense need for a reliable and authentic identical twin identification system. Since many twin detection methods rely on factors such as fingerprints, palm prints, speech recognition, iris, retina, mouth, ears, skin color, etc., and need significant processing time, the proposed approach bases its twin recognition on facial picture analysis. The principal objective is to create an accurate facial recognition system to identify identical twins implicated in criminal prosecution. The present proposed method is the result of an attempt to incorporate identical twin face recognition to overcome the limitations and challenges referred to Refs. 2 and 8– 10.

### 1.2 Statement of the problem

The genetic similarities between monozygotic twins present a distinctive challenge in forensic science ^11^: when conventional identification methods fail to differentiate between identical twins in criminal investigations, judicial outcomes become highly unpredictable, potentially resulting in wrongful convictions. According to a previous study, the global twin birth rate has increased by one-third on average over the last 40 years, accompanied by an increase in crime. Research by Rehkha et al. ^12^ documents this trend, noting that twin births have grown from 18.9 to 33.3 per 1000 births. This presents unique challenges for forensic identification, as genetic similarities between identical twins can create substantial difficulties in criminal proceedings. When one twin is involved in a crime, the judicial outcome becomes highly uncertain. Therefore, it is important to avoid making mistakes when using biometric techniques for identification to avoid convicting someone innocent. This approach achieves high identification rates and accuracy, which makes it suitable for real-world applications such as biometric security, access control (Tribuana et al. ^13^) and surveillance systems.

The current research extends previous work into identical twin recognition (Sanil et al. ^8, 14^) by shifting focus from global feature analysis to region-specific local feature extraction in 3D facial meshes. While earlier studies established foundational approaches for twin differentiation, this work addresses specific limitations by targeting previously underutilized facial regions. The core problem statement remains consistent between these studies, as it is foundational to the research area. However, the methodology and approach applied in this manuscript are significantly different, providing new insights and extending the previous research contributions. The previous research established promising frameworks for twin differentiation using global feature extraction in both 2D ^8^ and 3D ^14^ facial images. However, low-contrast parts of the face, such as the “Cheeks,” the “front of the head,” and the “jaw boundary,” potentially containing subtle morphological differences between twins were not focused. The features extracted from these locations can capture tiny changes between identical twins that were missed during global feature extraction. The prior work has been explicitly cited in the current manuscript to ensure proper acknowledgment of earlier contributions. The distinction between the problem statement and the new methodology applied in this work justifies the originality of the current study. By incorporating region-wise landmark analysis and advanced feature descriptor algorithms, we aim to capture minute morphological variations that escape detection through global methods, thereby significantly improving the accuracy and reliability of identical twin differentiation in forensic applications.

The proposed framework for this study was inspired by the factual case history of crimes committed by identical twins. The following is the case history of these crimes. This case series highlights the significant forensic challenges arising from the genetic similarity of monozygotic twins, demonstrating critical limitations in DNA-based criminal investigations across multiple jurisdictions.

• Case 1: Drug Trafficking in Malaysia (2003): A criminal case in Kuala Lumpur ^15^ illustrated the fundamental challenge of twin identification when police attempted to prosecute a drug trafficking offense. Two twin brothers were initially implicated based on drug-related evidence. However, judicial proceedings were ultimately compromised due to the inability of forensic DNA analysis to differentiate between genetically identical individuals, resulting in the release of both suspects.
• Case 2: Jewelry Theft in Berlin (2009): A high-profile theft of €6.5 million in jewelry from a prominent department store presented a classic twin identification dilemma. When sweat samples were collected and analyzed, investigators were unable to link either of the two identical twins to the crime. Consequently, both suspects were detained and subsequently released due to insufficient discriminatory evidence. ^16^
• Case 3: Potential Mistaken Identity in Terrorist Identification (2009): Nigerian security authorities repeatedly declared that Abubakar Shekau, the head of Boko Haram, had been killed, according to Asogwa. ^15^ But after a year, the recordings that appeared online revealed Shekau’s continued existence. ^17^ This raises the possibility that an innocent person was murdered inadvertently after being falsely recognized as Shekau (mistakable identity).
• Case 4: Sexual Assault Investigation in Marseille (2012): A complex sexual assault case involving six victims highlighted the forensic limitations of twin identification. Two identical twins, Elwin and Yohan, were arrested based on DNA evidence. The victims recognized the general perpetrator but could not definitively identify which twin was responsible, demonstrating the profound challenges in criminal prosecution involving genetically identical individuals. ^7^
• Case 5: Murder Investigation in Arizona (2011): A murder investigation was complicated by the presence of twin brothers, with authorities unable to conclusively establish which individual was responsible. It was thought that one of the two twin brothers had committed the crime. However, due to the lack of clear evidence supporting the suspect’s guilt from biometric verification, the case was prematurely closed.

The technology behind face recognition has changed over time. Researchers initially concentrated on using 2D facial photos, however, this method had issues with head orientations and changing illumination. To address these problems, scientists started investigating 3D facial recognition. Face details like depth and curves are captured in more detail by 3D photographs than by 2D ones. But there are disadvantages to 3D methods as well. They are not feasible for real-world applications since they need a lot of file storage and processing power. More recently, researchers have discovered that a superior answer is produced by merging 2D and 3D methods. While addressing the shortcomings of each technique separately, this combination (multimodal) strategy leverages the best aspects of both approaches. Results from the 2D-3D combined methodology are more accurate than those from each technique alone. The ND Twins and 3D TEC datasets have not been used together in many studies; thus, our proposed approach will make use of both to create a novel multimodal framework.

Hence, the proposed study aims to propose a novel and effective approach to developing an accurate 2D/3D facial recognition system based on local characteristics that mimic the method used by forensic experts to identify identical twins implicated in criminal acts, which are deemed difficult due to gene similarity, rendering standard forensic DNA testing ineffective using ND twin and 3D TEC datasets.

### 1.3 Paper organization

This work is divided into the following sections. A survey of similar works and the literature is included in Section 2. The contributions to the research are given in Section 3. Section 4 describes the method for analysing facial images to detect local facial characteristics using machine learning methods. Section 5 describes the research methods used in this study. Section 6 presents the results of this study. Section 7 contains the conclusions and future research directions.

## 2. Literature survey and related work

This section will review previous strategies to recognize identical twins, analyse their effectiveness, identify their drawbacks, and explain how the hybrid feature-based strategy addresses these shortcomings or builds on previous successes.

### 2.1 Background theory

It is well known in face recognition research that one of the biggest problems is differentiating similar faces, particularly lookalikes and identical twins. The task is further complicated by the striking similarity in the biometric prints of the faces of identical twins. To increase the accuracy of face recognition, numerous sophisticated algorithms and databases have been created over time and tested in a variety of scenarios. However, these initiatives have often failed to meet expectations. Improving the present automatic facial recognition systems are necessary due to the increasing number of identical twin births and their increased involvement in fraud and criminal activity. Since forensic face recognition techniques are made to comply with legal procedures, these systems must be integrated with them.

**Figure 2.**
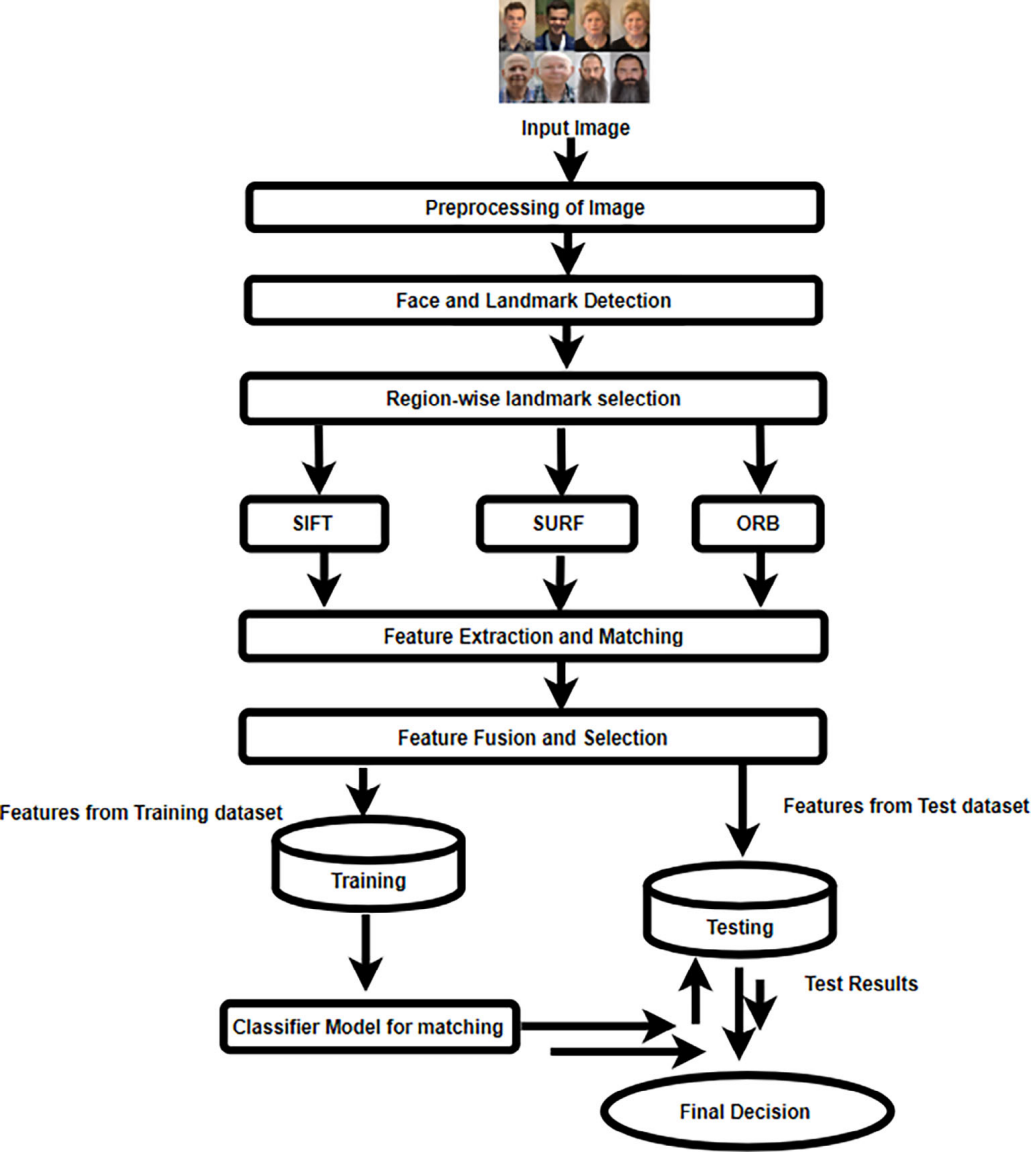
Representation of anthropometric landmark points on [a] Frontal facial image and [b] Side posture of the face. ^8^

**Face anthropometry:** The scientific examination of human body dimensions and proportions is known as anthropometry. Morphology is the study of forms in two and three dimensions, with an emphasis on the quantitative analysis of form and size that arises from the combination of geometry and biology. The face recognition system was developed in the 18th century by French biometrics researcher and police officer Alphonse Bertillon, who applied the anthropological method of anthropometry for criminal identification to create a break- through system. To describe human faces, qualitative and quantitative aspects were used. Figure 2 shows the anthropometric landmarks for the frontal facial picture (a) and the side profile of the face (b). The definitions for these landmark points are provided in Table 1. Table 1 provides a list of sample anthropometric landmarks on the face, along with their corresponding descriptions. These landmarks are used to derive various facial ratios. This comprehensive table provides definitions for key anthropometric landmark points, detailing their precise anatomical locations. These points are essential for accurate face measurements. The landmarks cover points on the face to provide a systematic approach to human face measurement. This study focuses on the analysis of facial characteristics and highlights the critical role of local regions ^18^ in face recognition systems.

**Table 1.** The anthropometrical landmarks of the face along with their descriptions. ^8^

Anthropometrical landmarks	Definition (Location)
Vertex (v)	The highest point on the head (Top of the skull, along the midline)
Trichion (tr)	Anterior hairline at the mid-line
Glabella (g)	The most prominent point between the eyebrows, above the nasal root (Junction of the frontal and nasal bones)
Nasion (n)	The point where the nasal and frontal bones meet (Junction of the nasal bones and frontal bones at the root of the nose)
Exocanthion (ex)	The soft tissue point located at the outer commissure of eye fissure
Endocanthion (en)	The soft tissue point located at the inner commissure of eye fissure
Palpebralesuperius (ps)	The highest point in the midportion of the free margin of each upper eyelid
Palpebraleinferius (pi)	The lowest point in the midportion of the free the margin of each lower eyelid
Orbitale (or)	The lowest point of the orbital margin (Lowest point of the eye socket’s bony rim)
Superaurle (sa)	The highest point of the free margin of the auricle
Subaurale (SBA)	The lowest point on the free margin of the ear lobe
Subnasale (sn)	The point where the base of the nasal septum meets the upper lip (Junction of the columella and the upper lip)
Sellion (se)	The deepest point of the saddle-like depression at the root of the nose (Between the nasal bones and frontal process of the maxilla)
Pronasale (prn)	The most protruding point of the nose tip (Anterior-most point of the nasal tip)
Alare (al)	The most lateral point of the nasal aperture (Lateral edges of the nostrils)
Labiale inferius (li)	The most anterior point on the vermilion border of the lower lip (Midpoint of the lower lip’s red border)
Labiale superius (ls)	The most anterior point on the vermilion border of the upper lip (Midpoint of the upper lip’s red border)
Cheilion (ch)	The point located at the corner of the mouth, specifically where the upper and lower lips meet laterally (The angle formed by the meeting of the upper and lower lips)
Stomion (sto)	The point of intersection between the upper and lower lips when the mouth is closed (Midpoint where upper and lower lips meet)
Gnathion (gn)	The lowest point on the mandibular symphysis (Lowest midpoint of the chin)
Gonion (go)	The most lateral and posterior point of the mandibular angle (Outer corner of the jawline where the jaw turns upward)
Pogonion (pg)	The most anterior point on the chin (Most forward point of the mandibular symphysis)
Tragion (t)	The most superior point of the tragus of the ear (Top of the cartilaginous projection in front of the ear canal)

### 2.2 Literature review on feature-based face image analysis for identical twin recognition

Over the last decade, researchers have made important breakthroughs in the field of face recognition of identical twins. Several researchers investigated various methods to this problem which are listed here. Biometric methods are of interest because they can distinguish between faces that are similar to each other, as mentioned in. ^19, 20^ The identification of criminals in the forensic sciences is one area in which face recognition systems are heavily utilized. Face recognition has several difficulties while being widely used and having many useful applications. ^21^ says that humans are naturally good at telling people apart based on their distinctive facial features. The human ability to measure a face for identification has been the main focus of facial recognition research. Consequently, there is a great deal of attention on creating new algorithms that imitate “human vision” to identify faces. Compared to studies on identification methods for regular people, there are far fewer studies on recognition technologies for identical twins based on various biometrics such as face, fingerprint, and iris. The study focuses significantly on work that discusses face recognition methods relevant to identical twins, similar faces, and their applications, more so to forensic aspects. Recent studies have shown that since identical twins share similar features due to their genetic similarity, the existing automated twin recognition system does not work well for monozygotic twins. ^22, 23^ Identical twins have similar features such as colour, DNA, eyes, ears, noses, and fingerprints, and therefore, differentiating the identical twins is a challenging task. ^24, 25^ The technologies currently used in the field of feature-based facial image analysis to identify twin faces, and similar faces are reviewed in the literature study that follows.

Moung et al. ^1^ proposed advanced techniques to address challenges in face recognition, including (i) automated face detection, (ii) variations in facial pose angles, (iii) occlusion impacts, (iv) diverse facial expressions, (v) aging-related changes, (vi) varying conditions of lighting, (vii) low image resolution, (viii) similarities between the identical twins and look-alikes, and (ix) other technical constraints. In addition to face recognition technologies, several biometric approaches have been proposed to improve the verification of the identification of identical twins.

Kukharev et al. ^2^ presents a review that covers the following topics: morphometry; unique cases in face image recognition, such as identical twins and lookalikes; both qualitative and quantitative techniques for determining parameters and assessing facial features; as well as multiple approaches using digital anthropometry. It also provides a brief history of the development of anthropometry in contemporary techniques and strategies that use computer technology to measure facial anthropometry. Primarily theoretical; lacks empirical validations.

Nafees et al. ^6^ used a “Gray-level co-occurrence matrix” along with a “Haar-Cascade classifier” to assess and discriminate against identical twins by studying RGB histograms of the eyes, lips, and face. One of their twin recognition investigations was carried out on a small dataset of just five pairs of twins. They added that this planned research on 3D face recognition can also be tested in controlled environments that can accommodate a variety of facial variances. A limited sample size is used.

Sanil et al. ^8^ introduced a unique method that combines human knowledge and machine learning to produce forensic evidence using 2D facial photos collected from the Web. To discover related facial traits, their solution uses machine learning approaches combined with hyperparameter tuning. For 2D facial photographs, the Euclidean distance is utilized to calculate the straight-line distance between two positions. In the case of curved surfaces, the geodesic distance is calculated by adding the linear distances between adjacent landmarks along the facial curvature between the two locations. Their technique achieved 78% accuracy with a small dataset. Low-contrast areas of the face, such as the “Cheeks,” “Forehead,” and “Jaw boundary,” were not focused, which could catch subtle changes between identical twins. A limited sample size is used.

Rehkha et al. ^12^ used different multimodal biometric techniques, such as hair wrinkles, facial marks, and facial features, using the PCA algorithm to discriminate between identical twins. When there are few biometrically comparable traits between twins, such as identical occipital hair whorls, they can be distinguished from one another more effectively by looking at their external ears and determining whether they are left- or right-handed.

Asogwa et al. ^15^ demonstrated an innovative technique that uses machine learning algorithms to identify identical twins and distinguish between two similar suspected faces belonging to distinct identities. This system is intended to aid in international criminal investigations. To further improve the system’s processing speed and recognition accuracy, more machine learning or deep learning-based approaches can be applied. Lacks specific performance metrics.

Biswas et al. ^21^ carried out various experiments under varying conditions to develop algorithms that mimic the human ability to discriminate facial features in twin identification. The results of this investigation lead to the understanding that the use of facial marks, along with the existing set of features, improves existing face recognition algorithms and machine performance. Their work recorded a precision of 78.825%. Limited feature integration strategy.

Phillips et al. ^22^ published a study on identical twins considering a time lapse of one year using the ND-Twins dataset. It was the first comprehensive analysis of twins’ faces utilizing three of the best submissions for the Multi-Biometrics Evaluation (MBE) COTFRS (commercial off-the-shelf face recognition system). It was revealed that due to sex, age, variations in lighting conditions, and expressions, the precision was greatly reduced. They also presented performance curves and error rates for various algorithms of face matching for differentiating identical twins under variable situations, which includes even those images taken with a time-lapse of one year and differentiating by gender and age. Performance degraded with lighting and expressions time lapse. Paone et al. ^26^ considered testing 7 different algorithms under various conditions for face recognition. Performance is assessed considering the four covariant: (i) age, (ii) gender, (iii) expression, and (iv) illumination. Their results revealed that they needed studio-like ideal conditions concerning illumination and facial expression with images acquired within a time-lapse of one year (not 2 years apart). It also showed that the performance of the algorithms was not affected by age and sex. The result ranged from 4.1 to 17.4 % as the best equal error rate.

Mousavi et al. ^27^ suggested a modified SIFT (M-SIFT) method in conjunction with crowdsourcing to discriminate between identical twins. They divided each facial image into five regions: eyebrows, eyes, nose, mouth, and face curves. Of these regions, the face curve was found to be the most important feature to differentiate between identical twins. Using this technique, 650 pictures were gathered in total, 115 identical twin pairs and 120 nonidentical twin pairs. As demonstrated by the test results, the lowest Equal Error Rate (EER) to identify identical twins was 7.8% for the full image, 8.1% for frontal images exclusively, and 10. 1% for PAN motion images. However, the facial region landmark detection (FRLD) method was unable to identify the landmark regions.

Sudhakar et al. ^28^ developed a fusion-based technique to differentiate identical twins. Principal component analysis (PCA), Gabor distance between face components, local binary patterns (LBP), and histogram-oriented gradients (HOG) were used in this study to extract features, which were then merged. Based on the scores produced by this fusion, twins were found. The best features were chosen using particle swarm optimization, and the images were trained and tested using a support vector machine (SVM). Compared to earlier techniques, this method produced greater precision and required less processing time. However, only photos with different faces and stances were considered; realistic photos were not.

Afaneh et al. ^29^ introduced a two-level decision process-based technique for identical twin recognition. They combined a CNN with fusion at the score, feature, and decision levels to increase accuracy. The study used ND TWINS-2009-2010 and traditional FERET data and used feature extractors, including PCA, LBP, and HOG. The results of the experiment demonstrated that the multimodal biometric system outperformed the unimodal systems in recognition. Under regulated illumination, the system achieved an Equal Error Rate of 2.2%, while for neutral expressions, it achieved a rate of 2.7% for identical twin recognition. Performance was highly dependent on controlled lighting.

Ahmad et al. ^30^ to distinguish between identical twins put forward a deep neural network. They used triplet loss to implement two different CNN models. Even powerful deep networks find it difficult to recognize identical twins with a precision of 87. 20% that they require.

Nahar et al. ^31^ applied a Transfer Learning approach in their study that incorporated geometric and photometric features. The authors also evaluated two networks trained with VGG-16. The integration of geometric and photometric features resulted in a precision of 98%. However, additional imaging data of identical twins is required. Four sets of twins, each recorded in 17 distinct positions, are included in Google Data. Photometric characteristics alone yielded an accuracy of 96%. In addition, different transfer learning techniques can be integrated with other modalities such as speech, facial recognition, and palm prints.

Venkatesan et al. ^32^ worked on a combination of Mean Landmark Points (MLPs) algorithm, SVM, and 3D PCA to present a face recognition system. Then, it was observed that the use of a carefully selected training dataset, along with the application of an SVM classifier on extracted features, could significantly improve the recognition rate. It was also noted that a process of initially segmenting multiple regions of the face, followed by each region being classified separately, and thereafter performing a fusion, would enhance the accuracy. It is also observed that this accuracy can be improved by using a multiclass SVM.

Parde et al. ^33^ investigated the ability of people and a DCNN to discriminate between identical twins and faces that are similar from different angles. They evaluated 87 individuals' ability to determine whether two faces in a group belonged to the same person or someone else using photos taken from frontal to profile views. According to the study's findings, DCNNs are becoming increasingly accurate in difficult face-identification scenarios, which could help applications in security and forensic settings by fostering human-machine cooperation.

### 2.3 Literature review on Facial Image Analysis to Identify Identical Twins using 3D-TEC dataset

Sanil et al. ^14^ highlighted the challenges faced in distinguishing identical twins due to their genetic similarities. The system utilizes geodesic distance algorithms such as Dijkstra’s algorithm, the Fast-Marching method, and A* algorithms for GD computation on 3D images, which account for the curvature of the facial surface, providing a more accurate measure of distances between 3D facial landmarks using the 3D-TEC dataset. This approach achieved 90% accuracy on a limited dataset, but their reliance on small sample sizes raises questions about generalizability to larger populations. While their real-time geodesic distance computation showed promise, the research failed to address computational complexities when scaling to larger datasets.

Vijayan et al. ^34^ conducted pioneering work with 3D twin images using SIFT and ICP algorithms but struggled significantly with expression variations (Cases III and IV), revealing fundamental weaknesses in handling changes in facial expression between twins. They used 3D facial landmarks including geodesic distance measurements, however their results showed that 3D techniques still had limitations, with recognition rates of about 72%.

Cai et al. ^35^ Pre-ResNet variants with multiscale triplet loss supervision reached 94.07% accuracy, but their approach depends critically on precise detection of the nose tip and pupils. This creates a significant vulnerability point, as recognition accuracy deteriorates substantially with even minor landmark detection errors. To obtain state-of-the-art recognition results, Quy et al. ^36^ introduced a SIFT-based method that achieved 84.6% accuracy but required parameter adjustments specific to the template database, limiting its adaptability to new datasets and real-world scenarios where parameter optimization may not be feasible. Required parameter tuning for the specific database.

Kim et al. ^37^ proposed a 3D face recognition system that utilizes a deep CNN. VGG- Face is a network that is optimized for 3D data after being pre-trained on 2D face images. The adaptation of VGG-Face showed substantial performance degradation (dropping to 80%) when handling expression variations, indicating limited robustness to emotional expressions that would be common in real-world applications. Compared to Cases I and II, the rank-1 performances in Cases III and IV were quite low (79.9% to 81.3%).

Al-Osaimi et al. ^38^ introduced an innovative method for expression-invariant 3-D face recognition, which uses the rotation-invariant and adjustable integral kernel (RAIK) technique to create key points for matching. The RAIK approach achieved 89% accuracy with improved expression invariance but relies on a heuristic graph search that introduces unpredictability in edge cases and potentially limits computational efficiency for large-scale deployment.

Li et al. ^39^ presented multiscale and multicomponent local normal patterns (MMSMC- LNPs), a unique approach for 3D facial recognition. MMSMC-LNPs approach with weighted sparse representation reached 95% accuracy but required a specialized training set, raising concerns about performance consistency across different demographic groups and data collection conditions.

Gilani et al. ^40^ developed the first deep three-dimensional face recognition network (FR3DNet) trained in 3.1 million 3D facial scans of 100,000 identities. The largest data set was known as LS3DFace. It incorporates information from several difficult public datasets, such as ND-2006, FRGC v2, Texas-3D, Bosphorus, GavabDB, BU4D-FE, CASIA, BU3D-FE, UMBDB, and 3D-TEC. FR3DNet, despite achieving 98% precision, depends on complex 3D face-dense correspondence for data augmentation that cannot guarantee identity closure properties. This introduces label noise that may compromise the model’s reliability in forensic applications where certainty is paramount.

Dutta et al. ^41^ presented a unique mathematical model to break down images in the range face into complementary components. The multistage approach with genetic algorithms and CNN classification suffers from excessive complexity, making practical implementation challenging and potentially limiting transparency in decision-making, a crucial concern for forensic applications.

Chen et al. ^42^ investigated the hybridization of feature extraction approaches by mixing SIFT with deep learning architectures. Their findings suggested that hybrid techniques could detect small changes between identical twins more effectively than single-method approaches.

**In summary,** it has been observed from reviews that existing facial recognition systems do not effectively identify identical twins. According to the current review level, facial recognition technology cannot successfully discriminate between identical twins. Due to their extreme genetic similarities, identical twins cannot be distinguished from each other using traditional forensic DNA tests, making the process nearly impossible. ^20^ Even for deep neural networks, it is difficult to identify identical twins based on facial photographs in an uncontrolled setting. ^30^ Despite the researchers’ efforts to create a more accurate and realistic twin detection system, there is still potential for advancement to overcome the aforementioned restrictions by taking into account a larger feature set to allow for fair twin discrimination. As a result, there is increasing interest in differentiating between identical twins using different biometric attributes and methods, particularly in forensic-related fraud and crime. As a result, the primary goal is to focus on low-contrast areas of the face, such as the “Cheeks,” “Forehead,” and “Jaw boundary,” and to analyze them using various approaches through a quantitative approach, which aids in identifying the performance measures that have a greater impact on the model. The ND -TWINS - 2009-2010 data set and 3D TEC were discovered to be the most difficult of the few data sets utilized for identical twin recognition, which is considered in our proposed approach.

## 3. Research contributions

The researcher’s goal is to create a novel system that uses machine learning to identify identical twins to support a criminal investigation. The system will have the following specific contributions.

1. To identify 468 landmarks utilizing the MediaPipe framework and selecting the region-wise landmarks for local feature extraction.
2. To generate feature vectors from identical twin images utilizing three feature descriptors, SIFT, SURF, and ORB, individually and in different combinations from local regions such as the nose, eyes, brows, and face curve, among others, taking into account region-wise local landmarks to increase the number of features used to identify the most distinctive regions and improve accuracy.
3. To extract 16*3 ratio-based features from the feature vectors produced using three different feature descriptors individually and in different combinations from local regions to capture minor differences that were missed in global feature extraction.
4. To analyse and compare features based on ratios using various machine learning algorithms to find facial differences more accurately for the recognition and verification process.
5. To validate and test this model using an ND-Twins dataset and the 3D TEC dataset to achieve comparable recognition performance and recommend this model for forensic applications.
6. To achieve accurate matching decisions for face recognition in cases of crime and fraud, despite varying facial expressions and pose variations in an unconstrained environment.

## 4. Methods

This study aims to present a new framework that can provide an optimal matching option for the identification of similar faces.

### 4.1 Overview of the proposed approach

Gathering the images from the ND twins and the 3D TEC database is the first stage. Pre-processing of the chosen image is required to recognize and crop the faces of each member of the twin pair. ^43^ The exact location of 468 points on 2D/3D facial photos was achieved using the media pipe framework. ^44^ Next, a region-wise selection of landmarks is performed using 468 landmarks to determine the number of significant points in the selected region, such as the nose, lips, eyes, eyebrows, and facial curves. These regions can serve as the basis for local feature extraction. The region-wise feature extraction is then performed by implementing the SIFT, SURF, and ORB feature descriptors on a single image. A list of key points with valuable image descriptions is achieved. Key points are areas of interest, which means that when a human sees an image at that particular moment, he observes certain aspects of that image. The term “Key points” refers to the specific spots of interest that a machine identifies when it examines an image. Descriptors are arrays or bins of numbers. These are used to describe the features; using these descriptors, we can match the two different images. The key points and image descriptors are computed for each detector. The system separates important points into two categories: mismatched points and matched points. The similarities between the two images are emphasized by the match points. Mismatched points serve as appropriate visual aids for illustrating image disparities. The region-matched and mismatched key points are used to calculate the ratios (16 * 3) concerning facial regions such as the eyes, eyebrows, nose, mouth, and face curves using SIFT, SURF, and ORB. The findings of these three descriptors are combined to create a fusion of characteristics. To determine how similar or distinct twin faces are depending on their attributes’, fused features are analysed employing machine learning models. A range of machine learning techniques, such as Nearest Centroid classifiers, eXtreme Gradient Boosting, Light Gradient Boosting Machine, and Support Vector Machine, were used to classify the data in the proposed experiment. The best machine learning models were chosen by comparing the efficacy of different models for a data set using the lazy-predict package. Comparison research has been illustrated using metrics such as the area under the curve (AUC), false positive rate (FPR), and true positive rate (TPR).

**Figure 3.**
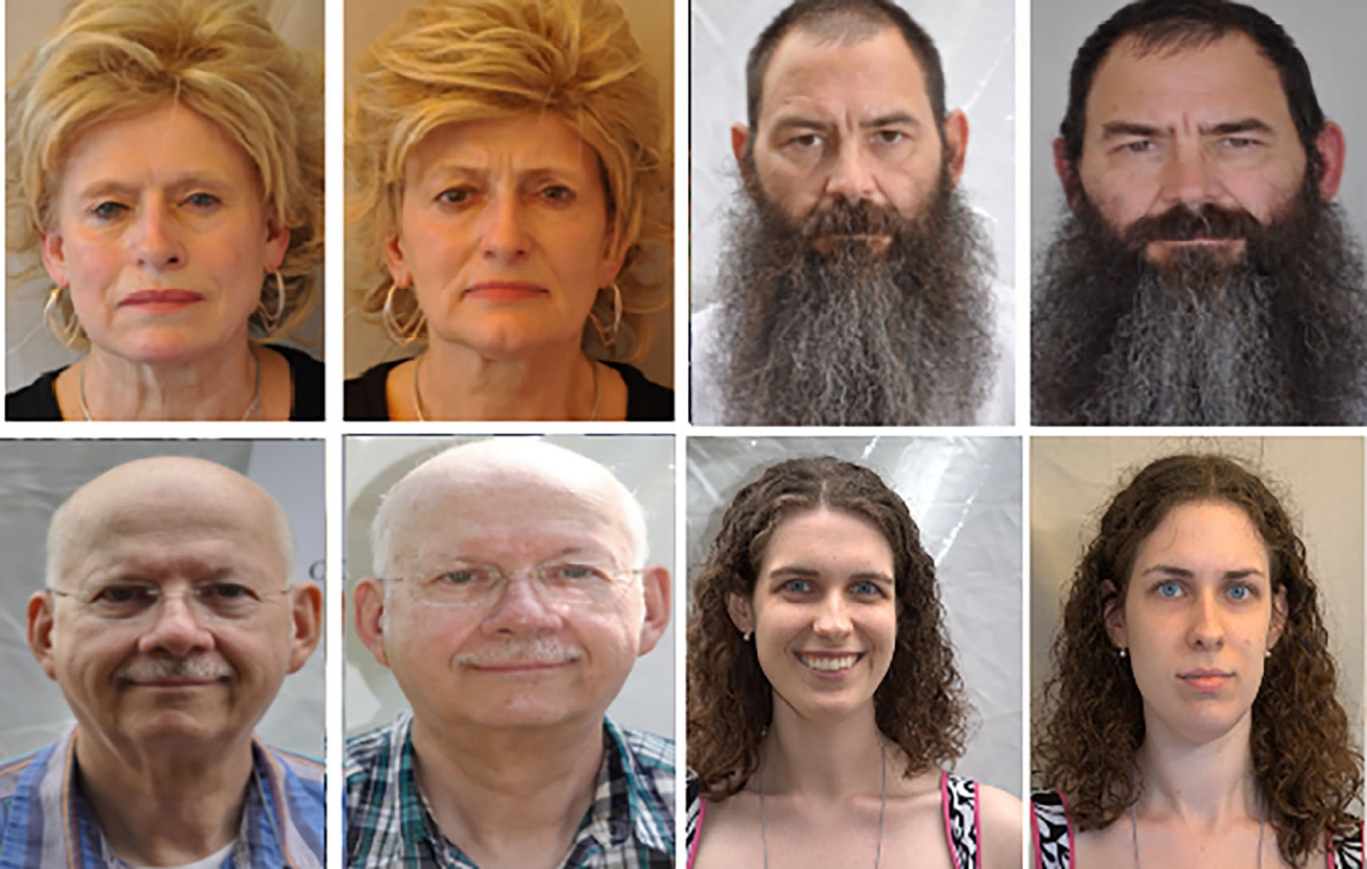
Schematic illustration of the Local feature extraction approach.

The proposed facial recognition system is divided into several steps, including pre-processing, face and landmark identification, extraction of features, and fusion, classification, and making decisions. Figure 3 displays the block diagram for the proposed methodology.

## 5. Research methods

The following are the methods used to test the feasibility of the proposed plan.

### 5.1 Data collection and validation

The primary step in facial image analysis is image acquisition. Data collection is done in the initial stage of the research before achieving the objective. The details of the data sets required in the proposed method are expressed below. In the proposed study, **ND-TWINS-2009-2010 Dataset** ^45^ and the 3D Twins Expression Challenge (3D-TEC) data set ^46^ were used.

**Figure 4.**
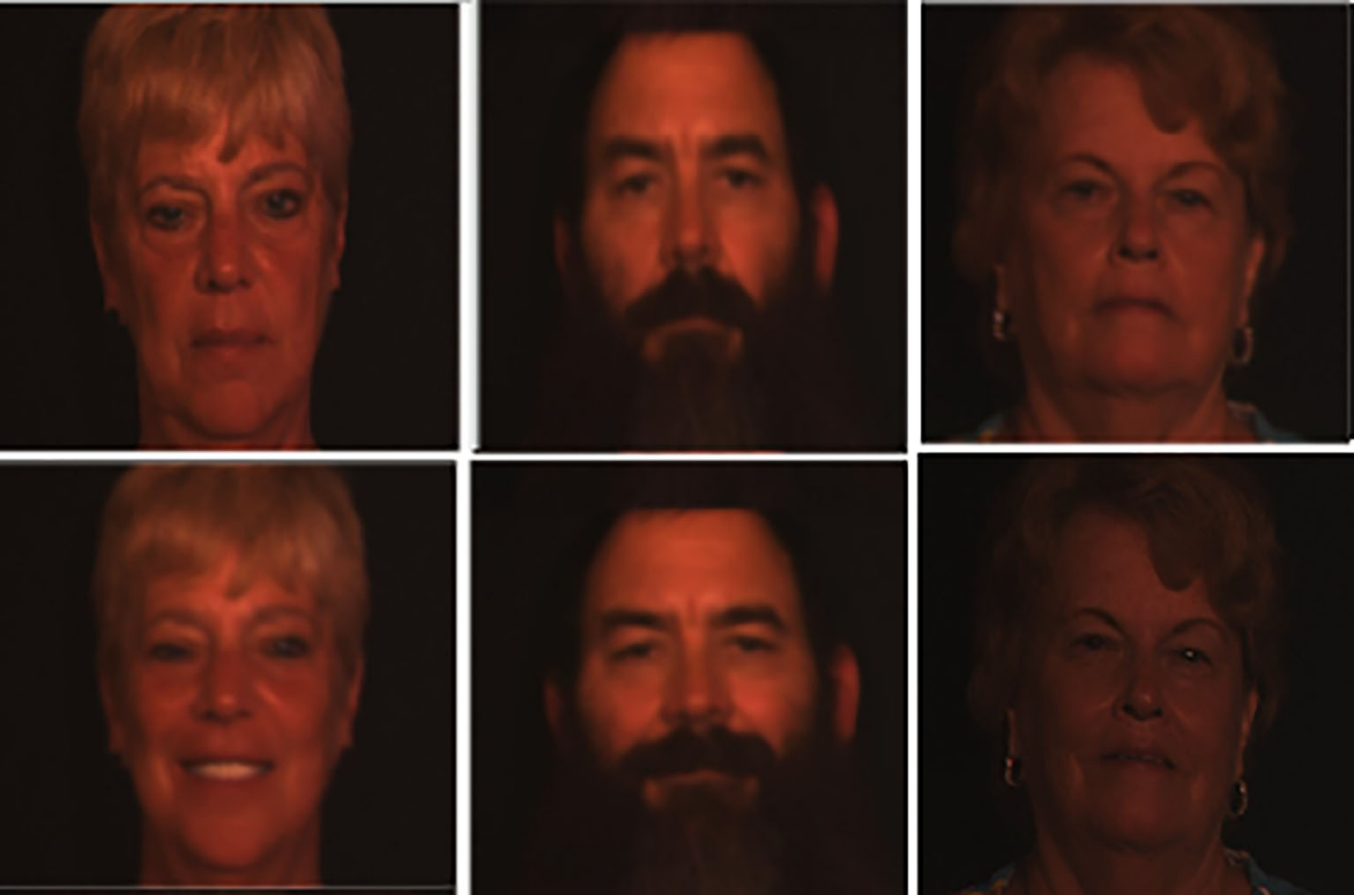
Sample facial images of ND-TWINS dataset. ^45^

**Figure 5.**
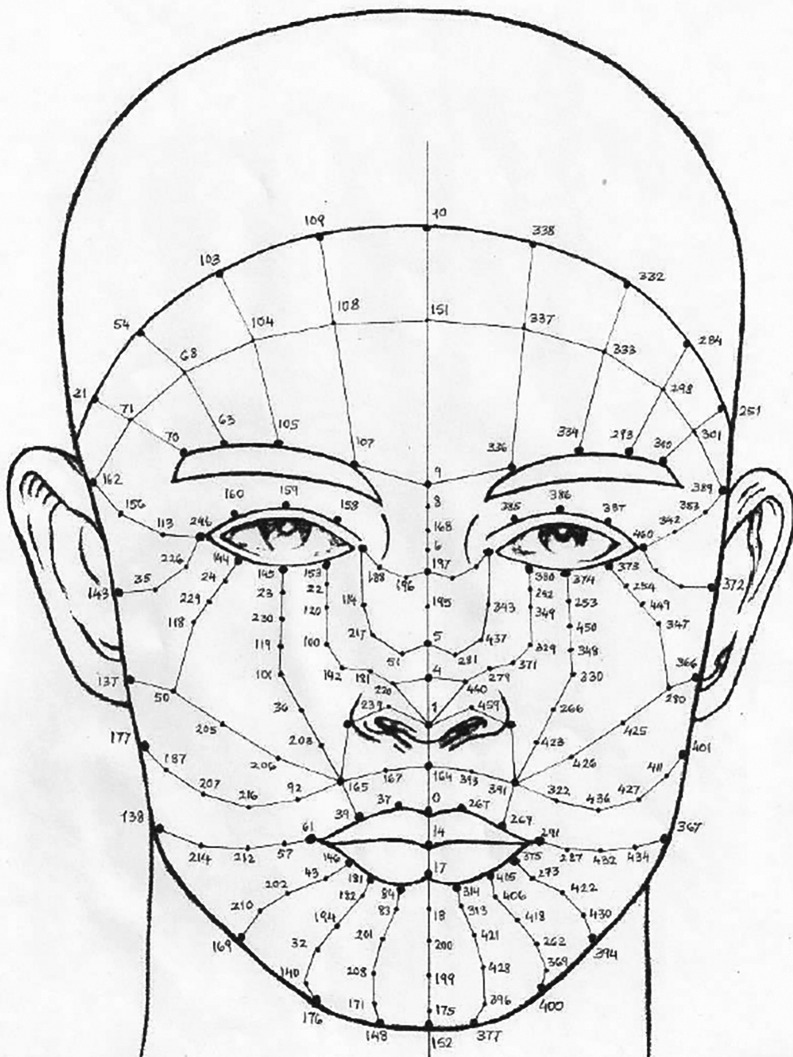
Sample facial images of 3D TEC dataset. ^46^

1. **ND-TWINS-2009-2010 dataset** ^45^ **:** Twins Days Festivals in Twinsburg, Ohio in 2009 and 2010 provided 435 participants with their faces captured in 24,050 colour images for the ND-TWINS-2009-2010 data collection. This collection contains images of pairs of siblings, fraternal twins, and identical twins in a variety of positions and lighting settings. Photographs were taken in natural light in both “indoor” and “outdoor” situations where a tent was used. The face yaw was measured in 45° steps, from -90 to +90° (frontal = zero degrees). This data set contains frontal face images for each subject in a neutral expression. The resolution of the images was 3456×2304 pixels. An example is shown in Figure 4.
2. **3D Twins Expression Challenge (3D-TEC) Data Set (Data type: Face 3D and Size=1.5 GB)** ^46^ **:** This is a Twins Days dataset, which contains 3D face scans of l07 pairs (total of 214 subjects) of twins, with neutral and smiling scan expressions taken for each subject. Although it is 10 times smaller than FRGC v2.0 data set, this sample of twins with varying expressions are fairly representative. This database will support the advancement of three-dimensional facial recognition technology. An example is shown in Figure 5.

The data set’s picture representation format is JPEG and PNG compatible. Of the images in the data set, 20% was used for testing and 80% for training. The ND-TWINS and 3D TEC datasets were chosen for their high-quality photos, variety of facial features, and importance to the identification of monozygotic twins. The ND-TWINS dataset contains high-resolution 2D facial photos with lighting, position, and expression variations, making it ideal for testing feature extraction and classification methods. Meanwhile, the 3D TEC dataset has 3D facial scans, allowing for a more comprehensive analysis by including depth information, which improves robustness in difficult conditions.

### 5.2 Facial image analysis

**Figure 6.**
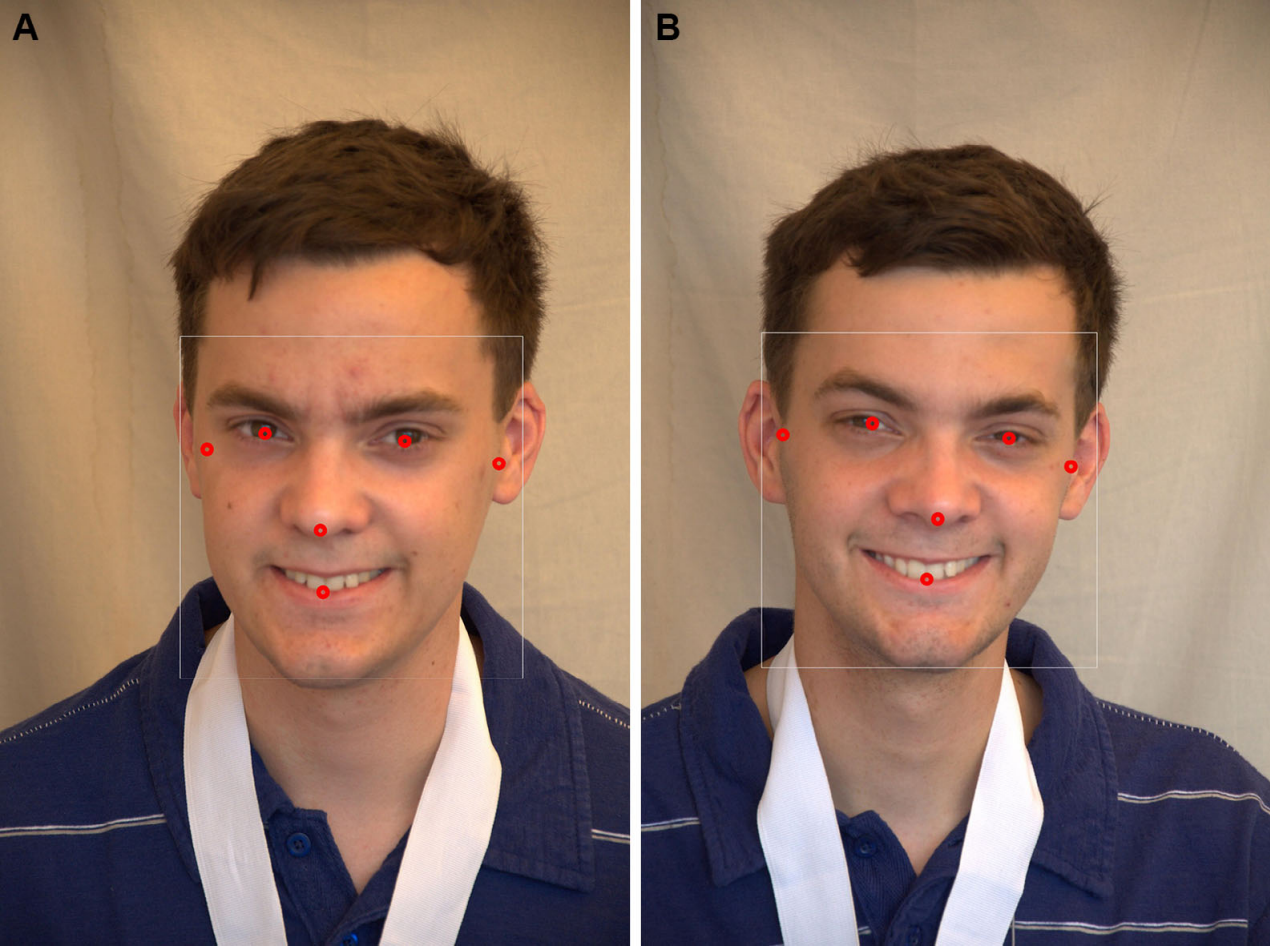
Manually mapped 468 facial landmarks.

The process of extracting useful information from pictures or videos of people’s faces using computer vision algorithms is known as facial image analysis. It analyses faces in images, videos, and real-time situations using computer algorithms and machine learning. Both human observers and computer systems can deduce a great deal from facial photographs, including age, identity, gender, race, emotions, and even attractiveness. The potential uses of machine learning-powered facial analysis methods have received much attention lately. Several important areas, including face detection, facial feature recognition, facial identification, and facial photo interpretation, are the focus of ongoing research in this field. The ability to identify and quantify the locations of facial feature points within an image is essential for performing face analysis tasks. The study of facial image analysis promises more advancements in comprehending and utilizing the complex information that human faces express as methods continue to develop. Figure 6 presents the results of a manual study of 468 anthropometric points in various facial regions.

**Figure 7.**
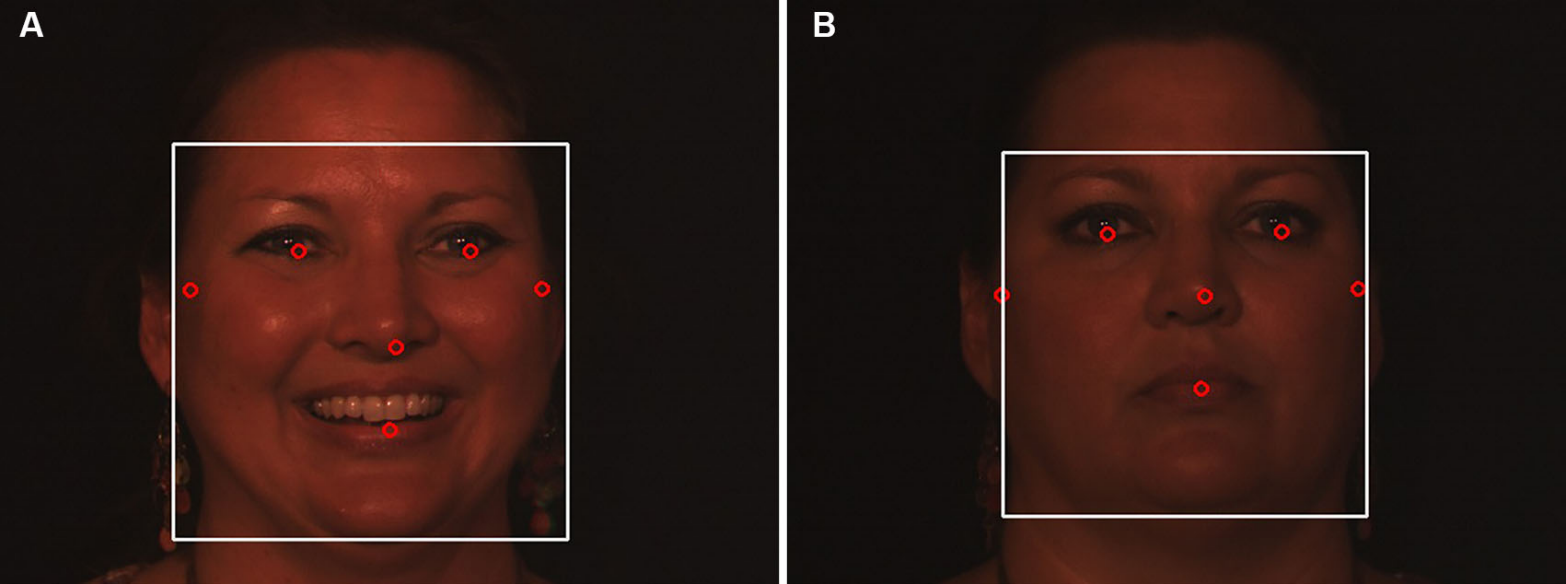
Face detection with 6 landmarks using MediaPipe framework considering the twin images from the ND twins dataset.

**Figure 8.**
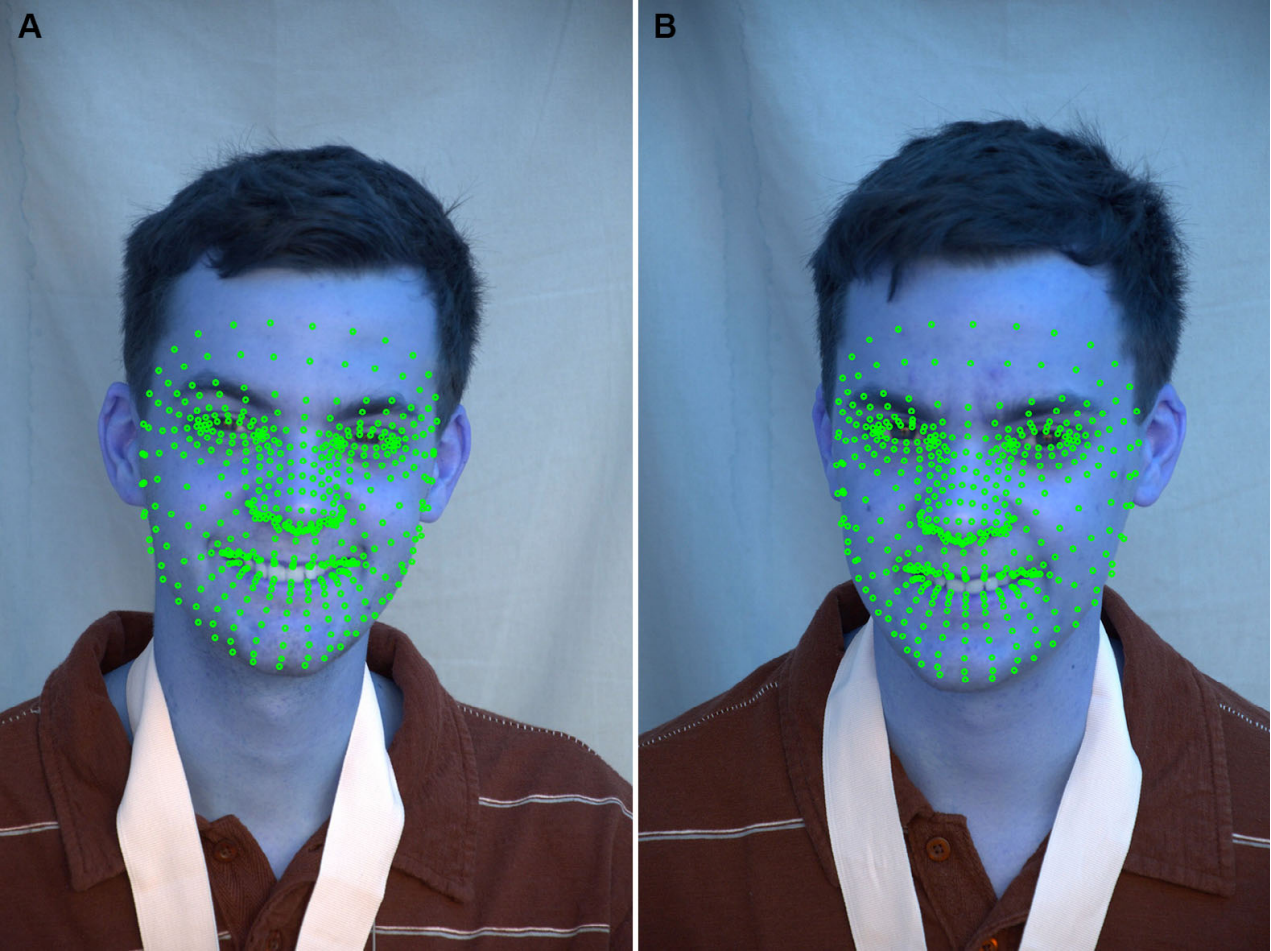
Face detection with 6 landmarks using MediaPipe framework considering the twin images from the 3D TEC Dataset.

**Figure 9.**
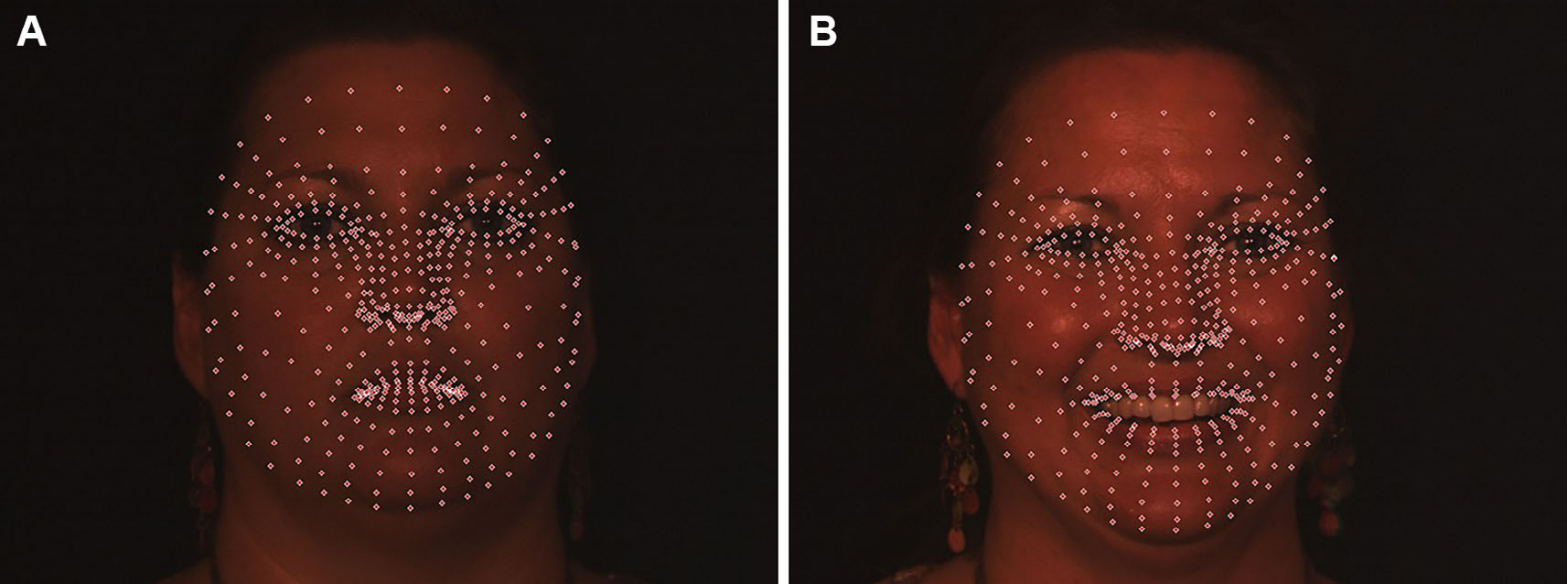
Landmarks detection using MediaPipe framework considering images from the ND Twins Dataset.

**Figure 10.**
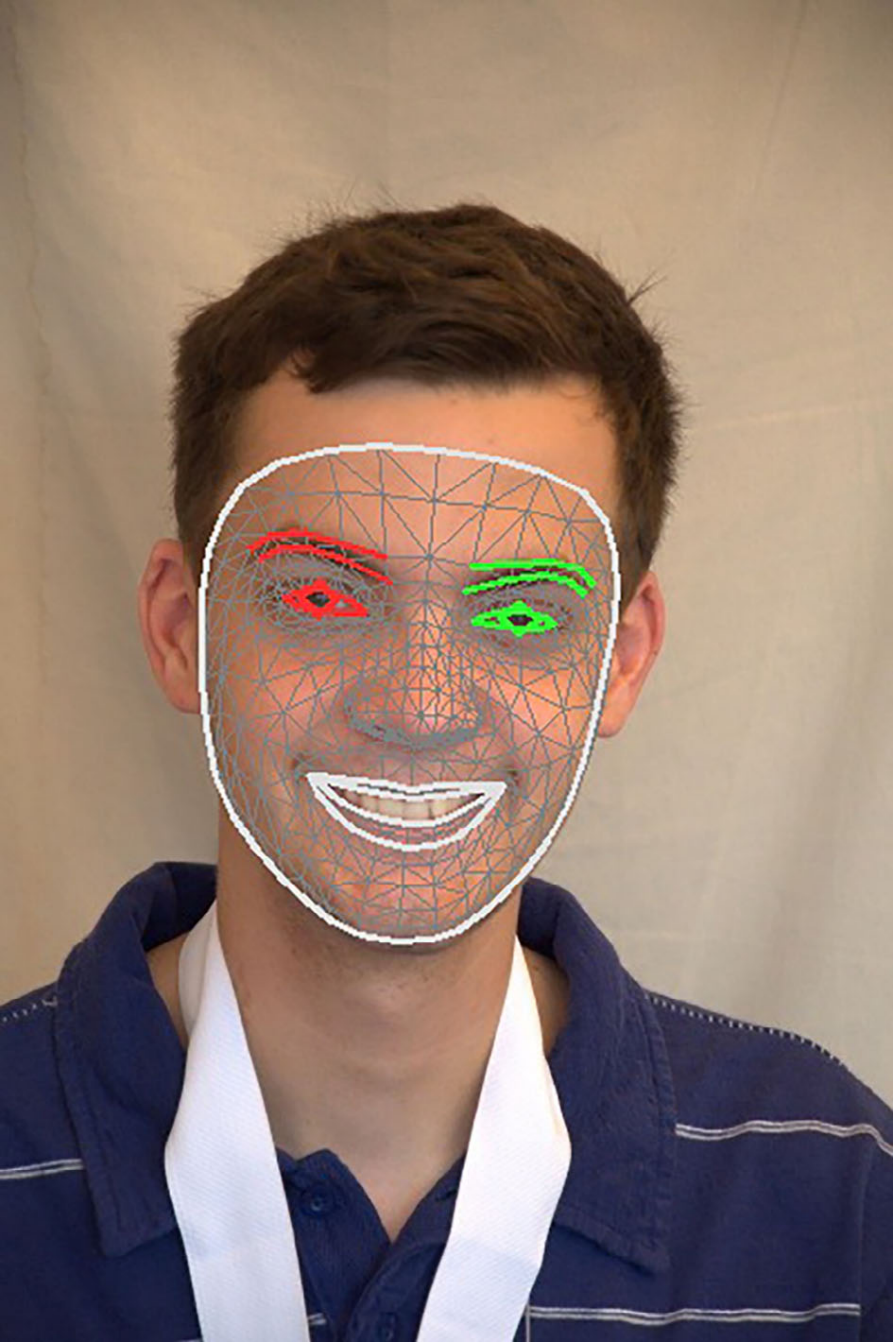
Landmarks detection using MediaPipe framework considering images from the 3D TEC Dataset.

1. **Face detection:** A human face can be found and identified in an image using a technique called a face detector, which produces a rectangle value or a bounding box as a result. The MediaPipe framework, created by Google, provides a quick and accurate way to create high-quality face detection models. In the proposed method, face detection is carried out using Media Pipe’s face detection model, which can detect faces in real time from either images or videos. In the proposed study, a human face image with six landmarks was detected, as shown in Figures 7, 8 using the ND Twins and 3D TEC datasets employing the media pipe face detection model.
2. **Landmark detection:** The practice of identifying and locating specific facial features is known as face marking. Google has developed a comprehensive architecture called MediaPipe [v0.8.11] ^47^ that allows the creation of multimodal applied machine learning pipelines (text, audio, and video). Its real-time human stance, hand, and face landmark identification capability is one of its standout characteristics. The MediaPipe framework is an open-source, cross-platform, face geometry solution library developed by Google for computer vision tasks that were used in previous studies to locate landmarks using 2D and 3D facial images. ^8, 14^ Estimated 468 3D face landmarks in real-time, even on mobile devices. The MediaPipe Python library uses a holistic model to detect multiple faces and 468 face landmarks in a 3D space. The 468 facial landmark identification model from MediaPipe displays every necessary point on a human face. The outcome was achieved by superimposing 468 landmarks on 2D/3D facial pictures from the ND twin and 3D TEC datasets and creating a face mesh from the collected 468 landmarks of the face. The well-known facial landmark identification techniques, such as MTCNN and Dlib, are more accurate, yet they fall short in some situations. Based on the literature, it is inferred that DLIB is relatively slower in terms of face and landmark detection capability compared to the MediaPipe framework when considering applications such as active face detection in live video or any other similar applications. ^48^ Although the face detection score was high for the MTCNN model, ^49^ the speed was low. The advantage of MTCNN is that it can identify occluded faces with some accuracy. However, MTCNN, with five landmark points was not sufficient to improve face recognition accuracy. The result of creating 468 landmarks using MediaPipe framework considering images from the ND Twins Dataset are shown in Figures 9 and 10. The (Midia Pipe software can be accessed here https://ai.google.dev/edge/mediapipe/solutions/guide). ^47^

**Figure 11.**
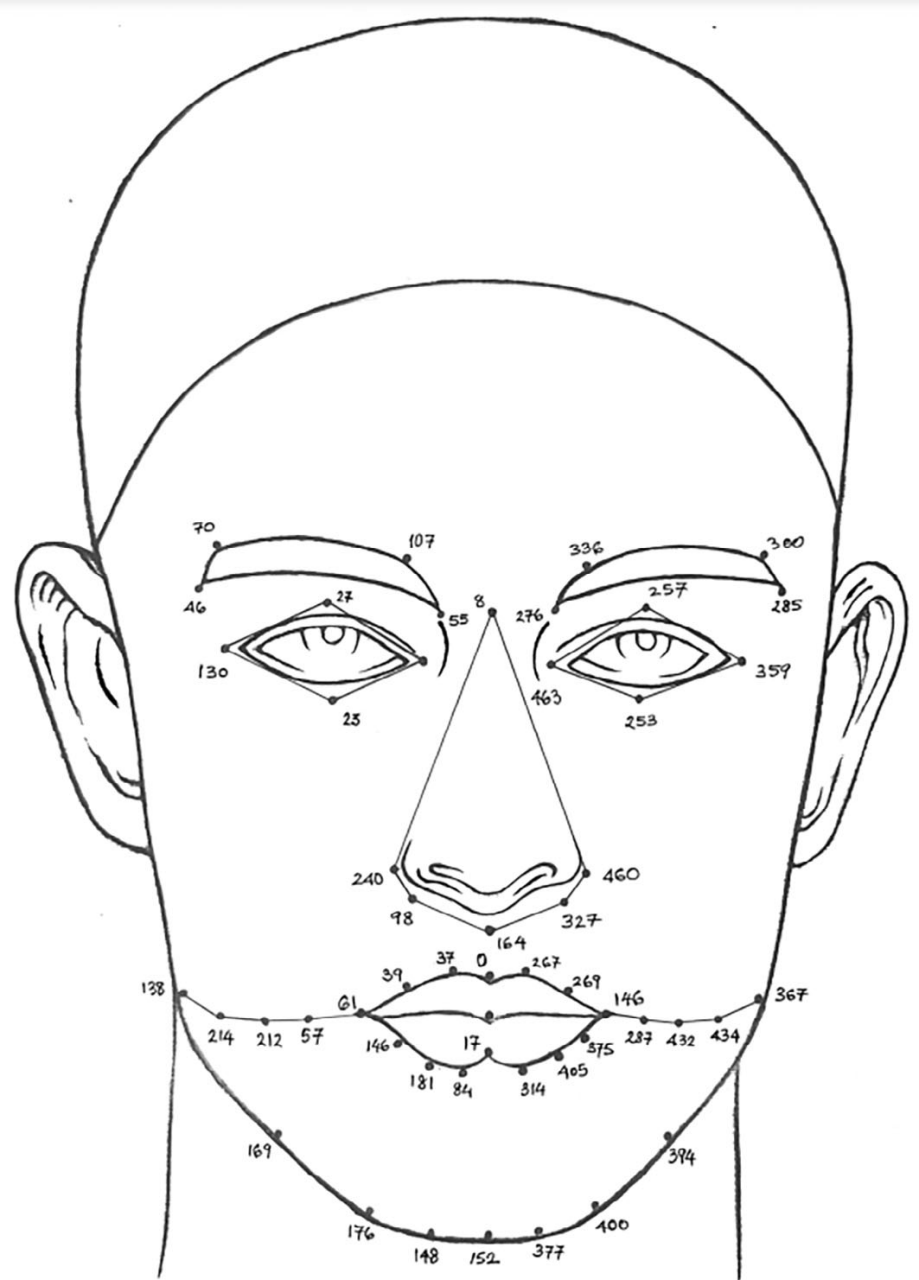
Superimposed 468 points, facial mesh is generated based on the extracted landmarks, and the facial regions are highlighted.

The output of superimposing 468 landmarks on facial image from the ND Twins dataset, creating a face mesh using the 468 facial landmarks extracted and highlighting the facial regions are shown in Figure 11.

In the current study, the MediaPipe framework face mesh solution was used to annotate the 468 landmarks in any uncontrolled condition (including the entire forehead region and additional points in the jawline region) from which significant landmarks were selected to obtain an increased number of variances that improved recognition accuracy. Forensic experts in criminal investigations rely only on anthropometry-based landmark measurements that are widely accepted in the court of law as statistical evidence; therefore, using a deep-learning-based approach is not acceptable. Hence, the MediaPipe framework, which works well even in real-time images, is used in this approach for landmark detection. This method is effective in real time for a range of illumination conditions, faces that are hidden, and faces that are of different sizes and orientations. The facial mesh topology provides more information than is needed; therefore, one can select the information that is needed. The MediaPipe framework provides more information than needed, and it is also possible to select only essential information with real-time performance. Applications currently implemented with MediaPipe include face detection, face mesh annotation, iris localization, hand detection, pose estimation, hair segmentation, object detection, tracking, and three-dimensional(3D) object detection.

3. **Region-wise landmark selection:** Using a variety of feature descriptors that concentrate on particular areas of an image to identify and characterize essential characteristics, region-wise landmarks are chosen to extract key points. When there are regions of an image that are more pertinent or hold more significant information than others, this method can be especially helpful. Only relevant landmark subsets were retained.

**Figure 12.**
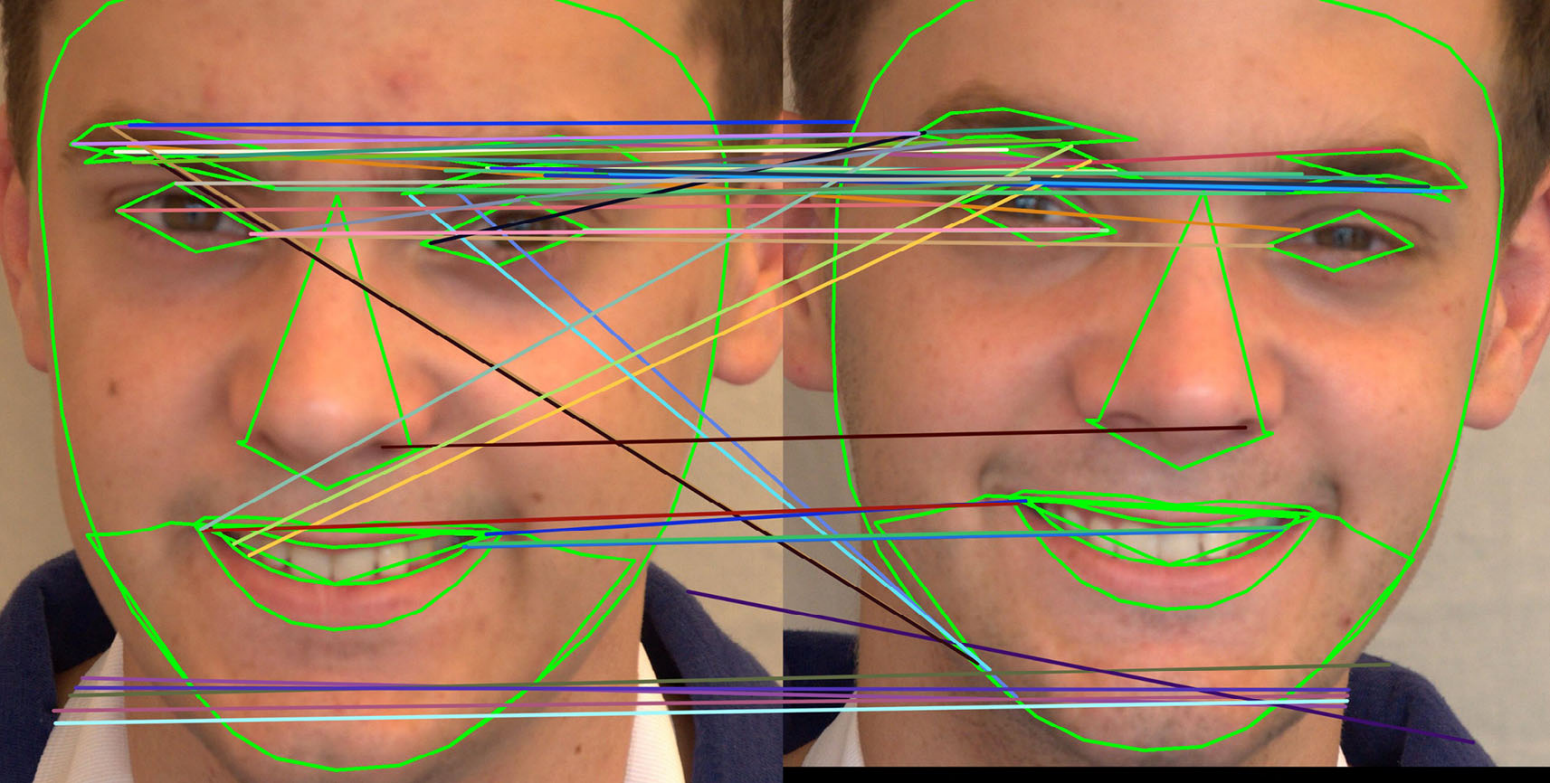
Region-wise selected landmarks.

A series of points found there can be used to identify each of the five regions:

• The **left eye** - [463, 257, 359, 253].
• The **right eye** - [130, 27, 243, 23].
• The **left eyebrow** - [276, 283, 282, 295, 285, 300, 293, 334, 296, 336].
• The **right eyebrow** - [46, 53, 52, 65, 55, 70, 63, 105, 66, 107]. The **nose** - [8, 240, 98, 164, 327, 460, 8].
• The **lips** - [61, 146, 46, 91, 181, 84, 17, 314, 405, 321, 375, 291, 65, 185, 40, 39, 37, 0, 267,269, 270, 409, 291, 78, 95, 88, 178, 87, 14, 317, 402, 318, 324, 308, 78, 191, 80, 81, 82, 13, 312, 311, 310, 415, 308].
• The **face curve** - [138, 214, 212, 57, 61, 14, 291, 287, 432, 434, 367, 379,400, 377, 152, 148, 176, 149, 138].
• The **face oval** can be accessed through points [138, 214, 212, 57, 61, 14, 291, 287, 432, 434, 367, 379, 400, 377, 152, 148, 176, 149, 138].
  Lotting these regions is necessary. The five components of a face, mouth, nose, eyes, eyebrows, and facial curvature are depicted in Figure 12. The region-wise selection of landmarks is utilized to determine the number of significant points within the selected area, including the mouth, nose, eyes, and eyebrows. These regions can serve as the basis for local feature extraction.

### 5.3 Region-wise landmarks-based feature extraction

The extraction of features is a fundamental task of face recognition algorithms. Because they transform visual information from an image into a numerical format that machine learning algorithms can compare and interpret, feature descriptors are crucial. The most important parts of an image are the highlights, dark areas, corners, and edges since they are always visible despite variations in the image’s form, brightness, or noise level. Identifying and describing key facial features that are useful for tasks such as facial landmark identification, facial emotion analysis, and face recognition is the aim of local feature extraction based on facial features. The objective is to isolate characteristics on the face that do not change with changes in lighting, posture, or expressions. Local feature-based extraction methods such as ORB (Oriented FAST and Rotated Feature Transform), Scale-Up Robust Features, Rotated BRIEF, and Scale-Invariant Feature Transform are widely used in computer vision for tasks such as object recognition, picture matching, and 3D reconstruction. The main goal of these techniques is to identify and characterize important spots or areas in an image that remain unchanged when subjected to various manipulations, including rotation, scale, and lighting adjustments. The proposed approach incorporates a local feature extraction technique that extracts features from local areas such as the eyes, nose, eyebrows, and face curve, among others, using well-known image matching techniques like SIFT, SURF, and ORB, both separately and in various combinations that utilize selected landmarks. A list of key points with valuable descriptions is achieved.

**Figure 13.**
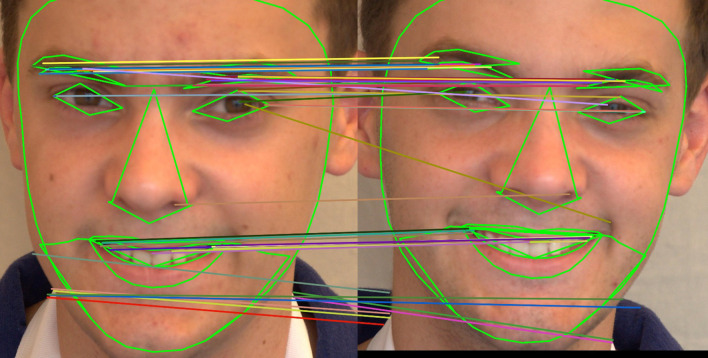
The application of SIFT feature descriptor to seven facial regions.

**Figure 14.**
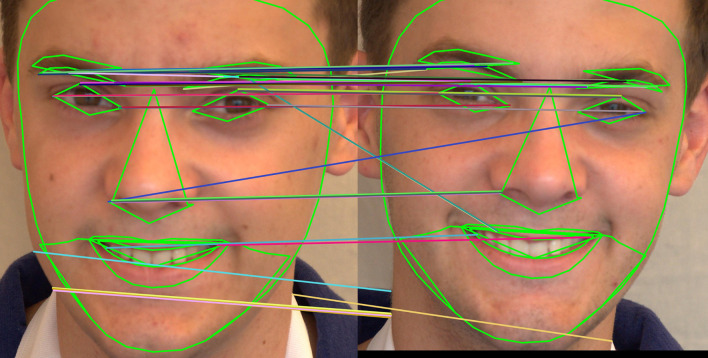
The application of SURF feature descriptor to seven facial regions.

**Figure 15.**
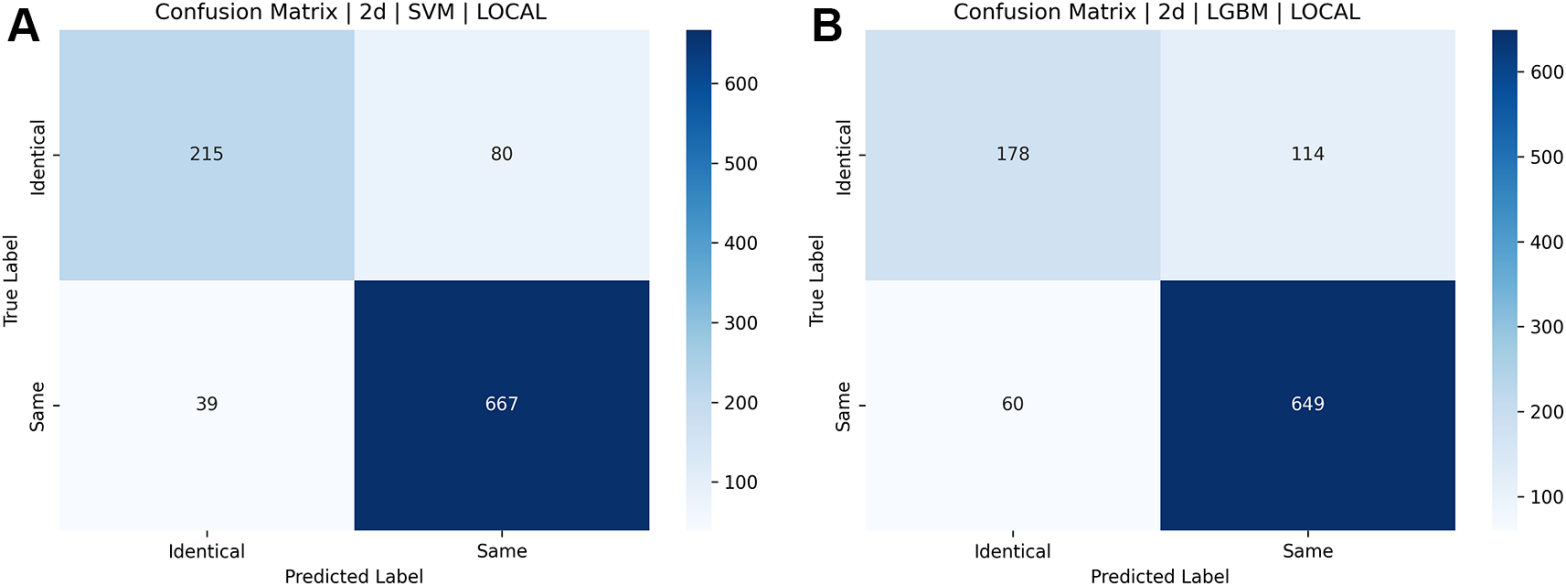
The application of ORB feature descriptor to seven facial regions.

1. **Feature mapping using the SIFT algorithm:** A computer vision method for feature identification and description is the SIFT (Scale-Invariant Feature Transform) algorithm. ^50^ The OpenCV is an open-source software that can be accessed here ( https://pypi.org/project/opencv-python /). Identifies recognizable focal spots or characteristics in a picture that hold up well under affine transformations, rotation, and scale adjustments. David Lowe created the algorithm in 1999. ^51^ Applications involving computer vision and image processing frequently employ it. Among the uses are object recognition, robotic mapping and navigation, 3D modelling, photo-stitching, recognition of gestures, individual wildlife identification, and match moving. The number of recognized key points (kp) and the key point descriptor (128), if present, dictate the size of a feature vector in the SIFT output. The SIFT algorithm has 4 basic steps:
  (a) Extrema detection in scale-space: The first part of the calculation looks for images of all sizes. It is accomplished by using a difference-of-Gaussian value to find interesting spots that are both scale- and orientation-invariant.
  (b) Key point Localization: Every possible place is fitted with an extensive model to determine location and scale. Points are selected using stability metrics.
  (c) Orientation Assignment: At least one orientation is assigned to every key- point position based on the gradient direction of the local image. To ensure these changes remain consistent, the picture data utilized for all subsequent operations is modified to reflect each feature’s given orientation, scale, and position.
  (d) Keypoint Description: Local picture gradients are measured close to every key point on the chosen scale. These are converted into a representation that permits notable variations in lighting and localized shape distortion. Figure 13 shows how the SIFT feature descriptors are applied to seven different facial regions.
2. **Feature mapping using the SURF algorithm:** SURF is a reliable and efficient method to match and detect key points of characteristics in computer vision & image processing. ^50^ The OpenCV is an open-source software that can be accessed here ( https://pypi.org/project/opencv-python /). The SURF framework is described in a paper by Bay et al. ^52^ To extract key points of the image feature, the algorithm makes use of a local invariant fast key point detector. This is a quick SIFT variant. It extracted the picture feature descriptor using a unique descriptor. It is a faster and more computationally sound method than the SIFT feature extraction method. 64 or 128-keypoint descriptions are produced. The primary attraction of the SURF technique is how quickly operators can be computed using box filters, allowing for real-time applications like object detection and tracking. The application of SURF feature descriptors to seven different facial regions is depicted in Figure 14.
3. **Feature mapping using the ORB algorithm:** Ethan Rublee et al. ^53^ created the feature detection and description method known as ORB (Oriented FAST and Rotated BRIEF) at OpenCV laboratories ^50^ in 2011 as a feasible and effective substitute for SIFT and SURF. The OpenCV is an open-source software that can be accessed here ( https://pypi.org/project/opencv-python /). Two ORB approaches, Binary Robust Independent Elementary Features (BRIEF) and Features from Accelerated Segment Test (FAST), work together to produce a key point detection system and a descriptor. It finds important spots using FAST first and then finds the top N points among them using the Harris corner measure. Figure 15 shows how the ORB feature descriptors are applied to seven different facial regions.

Using SIFT, SURF, and ORB, the regions chosen based on the landmarks are used to identify unique focal points in both images and calculate their descriptors, which represent the local image content surrounding those focal points. The two sorts of points that make up the SIFT/SURF/ORB algorithm’s essential points are matched and mismatched points. First, a twin pair’s similarity is indeed represented by the matched points. Every other key point, except for match points, is a local maximum; no two points share a similar description vector. These are referred to as mismatch points. The essential points that are out of order serve as an appropriate illustration of the twins’ differences. A significant piece of information on the primary difference between two identical twin photos can be found when the concentration of mismatched sites is large.

4. **Brute force matcher:** The similarity of the feature descriptors is usually measured by matching algorithms using distance metrics, such as cosine similarity, Hamming distance, and Euclidean distance. To determine the similarities between the features derived from the input image’s face and the reference image’s, one can use a matching algorithm to compare the two sets of features. The aim is to find a group of matches. The Brute-Force Matcher method was set up for the suggested investigation. Brute force matches are easy to use. The features of the first image are matched with that of another image using the Brute Force Matcher. ^54^ As the name “Brute Force” suggests BFMatcher will explore every option to identify the best matches. It begins by matching every descriptor from the first image to every descriptor from the second, then moves on to the second descriptor from the first image, matching every descriptor from the second image, and so forth. The best match could be identified by calculating the minimum distance needed, which is discovered by comparing the descriptors. In this case, matches are produced based on similar distances using the KNN (K-nearest neighbour) matching. ^55^ The matches need to be arranged according to their Euclidean distance from one another; the closer the matches are, the more accurate they are. The Euclidean distance is calculated between one of the selected key point descriptors and all key points in other images. This represents the closest distance between the key points in the two images. However, due to the presence of noise, occlusion, and other factors, not all matches will be correct. Hence, one needs to filter out the incorrect matches and retain only the good ones. This can be done using various techniques, such as the ratio test, RANSAC (Random Sample Consensus), or machine learning-based approaches. Based on a threshold, we only choose the best matches in this case. In this case, the distance between the two nearest neighbours is divided by their ratio to find the threshold. We first iterate over all the matches and then select only the matches for which the distance is less than 70% of the minimum length of the descriptors of the two images. We add these matches to a new list called ‘good matches’. This will help us further filter out irrelevant matches.
5. **Ratio based features:** A typical method in facial identification and analysis is to compute ratios of local features on the face and use them as input to machine learning models. A machine learning model can then be trained with these features to perform a variety of tasks, such as recognition of identical twins and expression analysis. Equations 1 and 2 are used to calculate the total number of matched and mismatched points compared to the total number of key points in the face.
$$\left(\text{Total match}\right)=\frac{\text{Number of matched points}}{\text{Number of total keypoints}}$$ (1)
$$\left(\text{Total mismatch}\right)=\frac{\text{Number of mismatched points}}{\text{Number of total keypoints}}$$ (2)

Equations 1 and 2 are utilized to compute the ratio of match and mismatch points on the seven landmark regions relative to the remaining areas in the face. Ratio-based features extracted using the matched and mismatched points on the seven landmark regions:

$$\left(\text{Ratio}\_\text{Match}\right)=\frac{\text{Total match points located in seven regions}}{\text{Total match points}\;\text{in the face}}$$ (3)

$$\left(\text{Ratio}\_\text{MisMatch}\right)=\frac{\text{Total mismatch points located in seven regions}}{\text{Total mismatch points in the face}}$$ (4)

How many matched points are found in the seven landmark zones that have been suggested is shown by the results of Equation 1. Quantifying the number of mismatched points inside the seven designated landmark zones is possible by utilizing the results of Equation 2. In case two identical twins have identical faces, the most distinguishable area between them is the one that has the greatest number of mismatched keys. The comparison of the quantity of mismatched key points in the selected landmark regions will be used to describe this. Two different feature sets were extracted and compared to find the best approach to recognizing faces. The first method uses the distances between the selected landmarks, while the second method employs ratios of distances. In this and other landmark regions, several features are proposed and extracted to evaluate the effectiveness of the face region in detecting identical twins. Equations 3 and 4 are used to obtain the following 14 features region-wise:

$$\left(\text{Ratio}\;1\right)=\frac{\text{Number of mismatched points on curve region}}{\text{Number of total matched points}}$$ (5)

$$\left(\text{Ratio}\;2\right)=\frac{\text{Number of mismatched points on Mouth region}}{\text{Number of total matched points}}$$ (6)

$$\left(\text{Ratio}\;3\right)=\frac{\text{Number of mismatched points on Nose region}}{\text{Number of total matched points}}$$ (7)

$$\left(\text{Ratio}\;4\right)=\frac{\text{Number of mismatched points on eyes region}}{\text{Number of total matched points}}$$ (8)

$$\left(\text{Ratio}\;5\right)=\frac{\text{Number of mismatched points on eyebrows region}}{\text{Number of total matched points}}$$ (9)

$$\left(\text{Ratio}\;6\right)=\frac{\text{Number of mismatched points on oval region}}{\text{Number of total matched points}}$$ (10)

$$\left(\text{Ratio}\;7\right)=\frac{\text{Number of mismatched points on curve region}}{\text{Number of total mismatched points}}$$ (11)

$$\left(\text{Ratio}\;8\right)=\frac{\text{Number of mismatched points on Mouth region}}{\text{Number of total mismatched points}}$$ (12)

$$\left(\text{Ratio}\;9\right)=\frac{\text{Number of mismatched points on Nose region}}{\text{Number of total mismatched points}}$$ (13)

$$\left(\text{Ratio}\;10\right)=\frac{\text{Number of mismatched points on eyes region}}{\text{Number of total mismatched points}}$$ (14)

$$\left(\text{Ratio}\;11\right)=\frac{\text{Number of mismatched points on eyebrows region}}{\text{Number of total mismatched points}}$$ (15)

$$\left(\text{Ratio}\;12\right)=\frac{\text{Number of mismatched points on oval region}}{\text{Number of total mismatched points}}$$ (16)

Similarly, using the landmarks identified by the media pipe structure, a total of (16*3) features are extracted in this study from other chosen facial landmark locations, such as the eyes, nose, mouth, and eyebrows, based on the key points generated by the SIFT/SURF/ORB algorithm. The 16 different measurements were selected to capture key facial proportions across different regions without collecting redundant information. By using three different feature detection methods (SIFT, SURF, and ORB), the system benefits from each method’s unique strengths - SIFT handles different scales well, SURF processes quickly, and ORB manages rotated faces effectively. Through careful testing of various measurement combinations, researchers found that using exactly 16 measurements with these three detection methods created the best results. These features extracted from the SIFT, SURF, and ORB are merged to form a comprehensive representation of the face, and the resultant feature vector is saved in the databases, which is used for similarity measurements.

### 5.4 Implementation details

The algorithm is implemented with Python 3.8 in the VS code framework and the power shell window. For automated face detection and landmark detection, existing models that are available in the “MediaPipe” framework are used. The experimental software environment was TensorFlow 2.10, ^56^ Python 3.11 ^57^ and the computer configuration was as follows: 11th Gen Intel(R) Core (TM) i7-12650H 2.30 GHz; 16.0 GB RAM; PCd AMD Ryzen 1600, 16GB RAM, RTX 4070; and Cuda11.3.1 with Cudnn8.2.1. Algorithm 1 explains the feature extraction process of the proposed approach. The data was split into training and test sets using an 80:20 ratio for all experiments.

Algorithm 1. **Ratio-based feature extraction.**

 
**Input:** A set of images from ND-Twins dataset and the 3D TEC datasets

 
**Output:** Ratio-based features extracted using SIFT, SURF, and ORB

**Procedure** compute_ratios():

 
**foreach** *image in image_set* **do**

 • Detect the face and compute the face bounding box.

 • Use MediaPipe to extract 468 landmarks.

 • Perform region-wise landmark selection.

 • Generate feature vectors using ORB, SURF, and SIFT separately and in combinations.

 • Obtain matched and mismatched key points from the selected regions.

 • Extract ratio-based features from matched and mismatched points.

 • Store all ratio-based features (16×3) in the database, both individually and in combination, obtained via SIFT, SURF, and ORB;

TensorFlow is an open source software library for high performance numerical computation that can be accessed here ( https://pypi.org/project/tensorflow-gpu/2.10.0/).

Python Software Foundation License, which is an OSI-approved open-source license, meaning it’s free to use, modify, and distribute that can be accessed here ( https://www.python.org/downloads/release/python-3110/).

The resultant ratio-based features obtained from the twin images are stored and then used for comparison through various machine learning tools such as support vector machine, light gradient boosting machine, XGBoost, and nearest centroid classifiers to find the mismatch.

### 5.5 Classification using machine learning algorithms

Giving an image a class through classification helps facial recognition. A class is then allocated to that similar group once the picture dataset is categorized based on the features that were retrieved from the photos. The test photos are assigned a class name by the twin recognition system after the features retrieved are compared with the training dataset. Train a classifier using the fused local features as input and a label indicating whether the faces belong to the same twin pair or not. To test the trained classifier model, extract the SIFT, SURF, and ORB features from a questioned image and enter the query object image. To identify the mismatch, the resulting ratio-based characteristics of the twin photos are saved and subsequently compared using a variety of machine learning techniques, including the support vector machine, light gradient boost machine, XGBoost, and closest centroid classifiers. Lazy Predict, a Python-based code, ^58^ is used to select the best machine learning models. In the proposed approach, four classification models were selected. It is useful for comparing basic models and determining which model performs best without any parameter adjustment. In this study, the four best classification models were chosen using the Lazy Predict Python program. SVM excels at finding the subtle differences between nearly identical faces and performs well even with a limited twin dataset. LGBM processes complex facial measurements quickly and efficiently, which is essential when analyzing multiple facial regions simultaneously. XGBoost helps manage inconsistencies in facial feature detection and avoids overfitting to specific twin examples, instead of learning true distinguishing characteristics.NC is useful for classifying data based on feature similarity, making it a simple yet effective approach. These classifiers were selected after evaluating their performance and suitability for the extracted features proposed compared to other traditional methods. The testing showed that these classifiers work better together than the other approaches we tested. Their combined abilities create a system that can reliably detect minor differences between twins under various real-world conditions. The accuracy, sensitivity, specificity, etc., were also measured using the ND twins and 3D TEC dataset to test the model’s performance. The Python module Scikit-learn is used to perform machine learning analysis ^59^ to classify facial images based on their similarity. The efficacy of the local feature extraction strategy for twin face recognition is tested using key performance criteria such as precision, recall, and F1 score.

### 5.6 Performance metrics for classification

The parameters of accuracy, precision, recall, F1 score, and area beneath the receiver’s operating characteristic (ROC) curve were used to compare and assess the suggested approach. A confusion matrix is an essential tool for determining a model’s benefits and drawbacks. The performance of a classification model in machine learning and statistics is evaluated using this table. The sklearn metrics module provides the Confusion Matrix, which is used to measure the number of accurate and inaccurate predictions in addition to other metrics such as precision, recall, and accuracy.

To compute all the measures above, the confusion matrix (CM) that includes true positives (TP), true negatives (TN), false positives (FP), and false negatives (FN) is shown in Table 2. The performance of classifiers utilizing supervised machine learning techniques is often evaluated using metrics obtained from the confusion matrix, which are mentioned below.

1. **Accuracy** is the model prediction given by the sklearn-metric module. It serves as a gauge for the model’s overall efficacy and a test of the data classification system’s performance. This was calculated using Equation (17).
$$\text{Accuracy}=\frac{\mathrm{TP}+\mathrm{TN}}{\mathrm{TP}+\mathrm{TN}+\mathrm{FP}+\mathrm{FN}}$$ (17)

**Table 2.** Confusion matrix.

	Actual values	
	Positive (1)	Negative (0)
Positive (1)	*TP*	*FP*
Negative (0)	*FN*	*TN*

Where TP and TN stand for correct predictions, FP and FN stand for wrong predictions.

2. **Precision** is the percentage of accurate positive predictions. Equation (18) is used to calculate it.
$$\text{Precision}=\frac{\mathrm{TP}}{\mathrm{TP}+\mathrm{FP}}$$ (18)
3. **Sensitivity/recall**, also known as the “true positive rate” (TPR), was computed by dividing the total number of accurate positive predictions by the total number of false and true positives. Equation (19) is used to compute it.
$$\text{Sensitivity}=\text{Recall}=\frac{\mathrm{TP}}{\mathrm{TP}+\mathrm{FN}}$$ (19)
4. **Specificity** was computed by dividing the total number of valid positive predictions by the sum of TP and FN values. This is called the “true negative rate” (TNR). Equation (20) is used to compute it.
$$\text{Specificity}=\frac{\mathrm{TN}}{\mathrm{TN}+\mathrm{FP}}$$ (20)
5. **The F1-score** is the harmonic means of recall and precision, where 1.0 represents the most significant score, and 0.0 represents the poorest score. This highlights both false negatives and false positives. This was calculated using Equation (21).
$$F1=\frac{2\times \text{Precision}\times \text{Recall}}{\text{Precision}+\text{Recall}}=\frac{2\times \mathrm{TP}}{2\times \mathrm{TP}+\mathrm{FP}+\mathrm{FN}}$$ (21)

### 5.7 Statistical analysis of feature distributions

To explore and validate the statistical significance of differences between the two classes (same person vs identical twin), a comprehensive statistical analysis was conducted on all features in the dataset used to classify whether two facial images represent the same person (label = 0) or identical twins (label = 1).

**Descriptive Statistics**: For each feature, descriptive statistics were computed separately for each class as shown in Table 3. These included Mean and Standard Deviation to describe central tendency and variability. Also 95% Confidence Intervals (CI) for the mean, calculated using t-distribution-based methods, to estimate the range within which the true population means is expected to lie with 95% certainty.

**Table 3.** Top 10 most statistically significant features that differ between same person (0) and identical twin (1) classes.

Feature	Mean (0)	Mean (1)	T-test p-value	Mann-Whitney p-value
ORB_NOSE_MIS_MATCH	0.0352	0.0720	1.21e-71	2.25e-93
ORB_L_EYE_MIS_MATCH	0.0352	0.0666	1.48e-59	2.99e-72
ORB_R_EYE_MIS_MATCH	0.0366	0.0678	3.84e-57	1.40e-66
SIFT_OVAL_FACE_MIS_MATCH	0.0661	0.1126	1.05e-51	8.47e-53
SURF_OVAL_FACE_MIS_MATCH	0.0361	0.0583	9.32e-45	1.23e-41
SURF_FACE_CURVE_MIS_MATCH	0.0312	0.0490	1.30e-40	4.99e-39
ORB_FACE_CURVE_MIS_MATCH	0.0455	0.0748	4.92e-38	2.06e-43
ORB_L_EYE_MATCH	0.1168	0.0861	5.11e-34	8.10e-23
SIFT_R_EYE_MIS_MATCH	0.0367	0.0553	5.15e-33	1.39e-35
ORB_LIPS_MIS_MATCH	0.0362	0.0564	1.04e-32	6.30e-40

**Tests for Statistical Significance:** To rigorously assess whether these differences were statistically significant, two tests were performed for each feature:

1. **Independent Two-Sample t-test:** This test evaluated whether the **mean values** of each feature differ significantly between the two classes. To accommodate unequal variances between groups, Welch’s t-test was used. A p-value < 0.05 was considered statistically significant.
2. **Mann-Whitney U Test**: As a non-parametric alternative, the Mann-Whitney U test was applied to compare the **rank distributions** of the features without assuming normality. This test is particularly robust to non-normal distributions and confirmed the t-test results.

**Figure 16.**
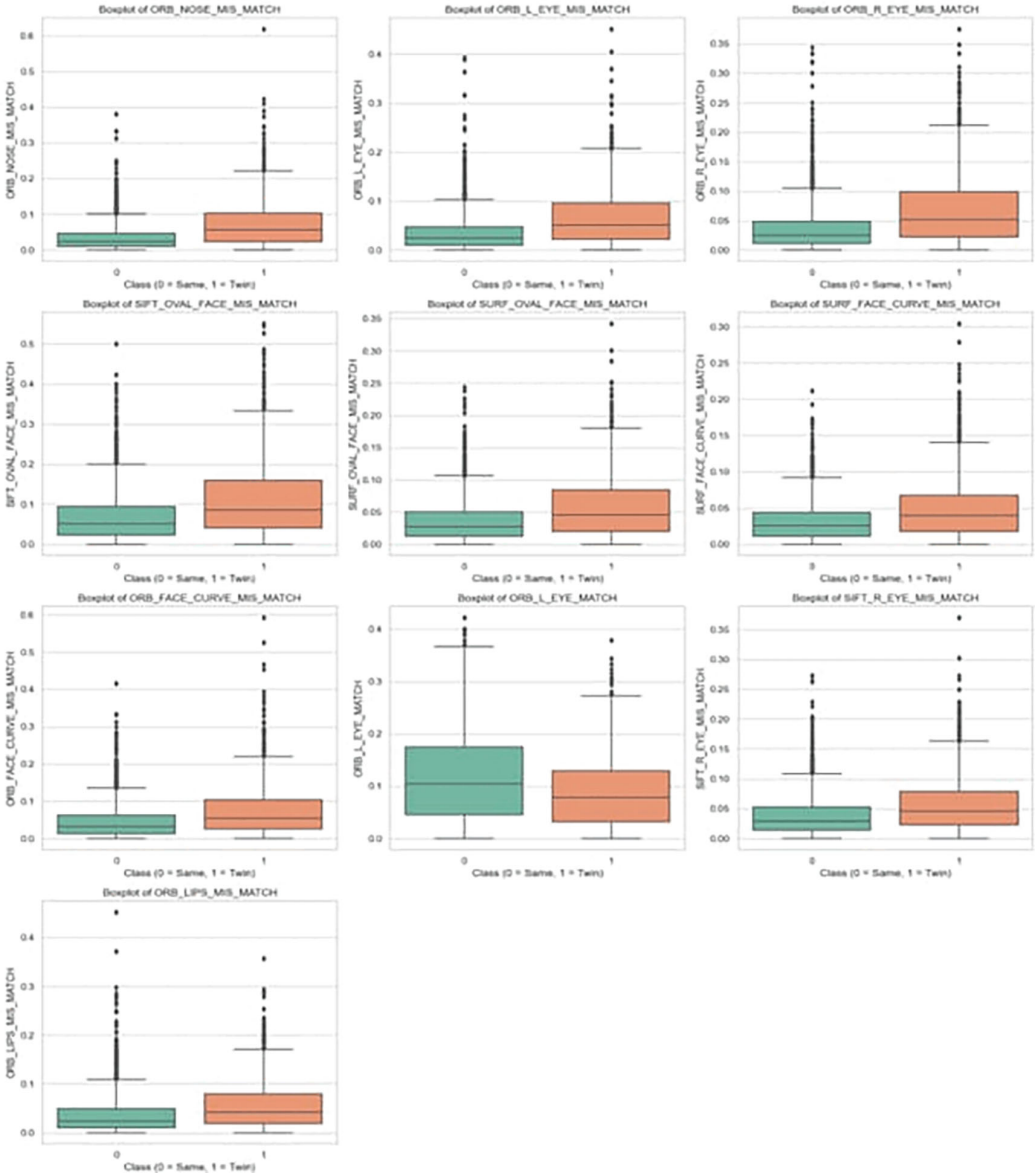
Boxplots for the top 10 statistically significant features.

The statistical tests revealed that several features exhibited **highly significant differences** between the classes, with p-values well below 0.001 in both t-tests and Mann-Whitney tests. The discriminative top 10 features are included. These features had both l **arge differences in mean values**, and **non-overlapping confidence intervals**, indicating strong separation between the two classes. Boxplots were generated for the top features show in Figure 16, visually confirming that features associated with mismatch scores tend to be significantly **higher for identical twins** than for the same person. This validates that the classifier can capture subtle but consistent biometric differences between these groups.

The incorporation of statistical measures and hypothesis testing provides strong evidence that certain facial features differ significantly between identical twins and the same person. These findings support the reliability of the selected features for classification tasks and justify their inclusion in any downstream machine learning models.

## 6. Results and Discussion

This section displays the results of face recognition using classification models using SIFT, SURF, and ORB feature descriptors, as well as other combinations of these feature descriptors. The effectiveness of these approaches is examined to determine their capacity to capture localized, fine-grained patterns for accurate classification. Training represented 80% of the data in this study, while testing represented 20%. A variety of machine learning algorithms and performance metrics are used to achieve the highest degree of precision. Simple fusion includes combinations of SURF+ORB, SIFT+SURF, SIFT+ORB, and SIFT+SURF+ORB combinations. The results of the experiment also indicate that a hybrid feature extraction method that incorporates the advantages of SIFT, SURF, and ORB can achieve a high recognition accuracy of 88% for the ND twin’s data set and 74% for the 3D TEC dataset when used with a Support Vector Machine.

**Figure 17.**
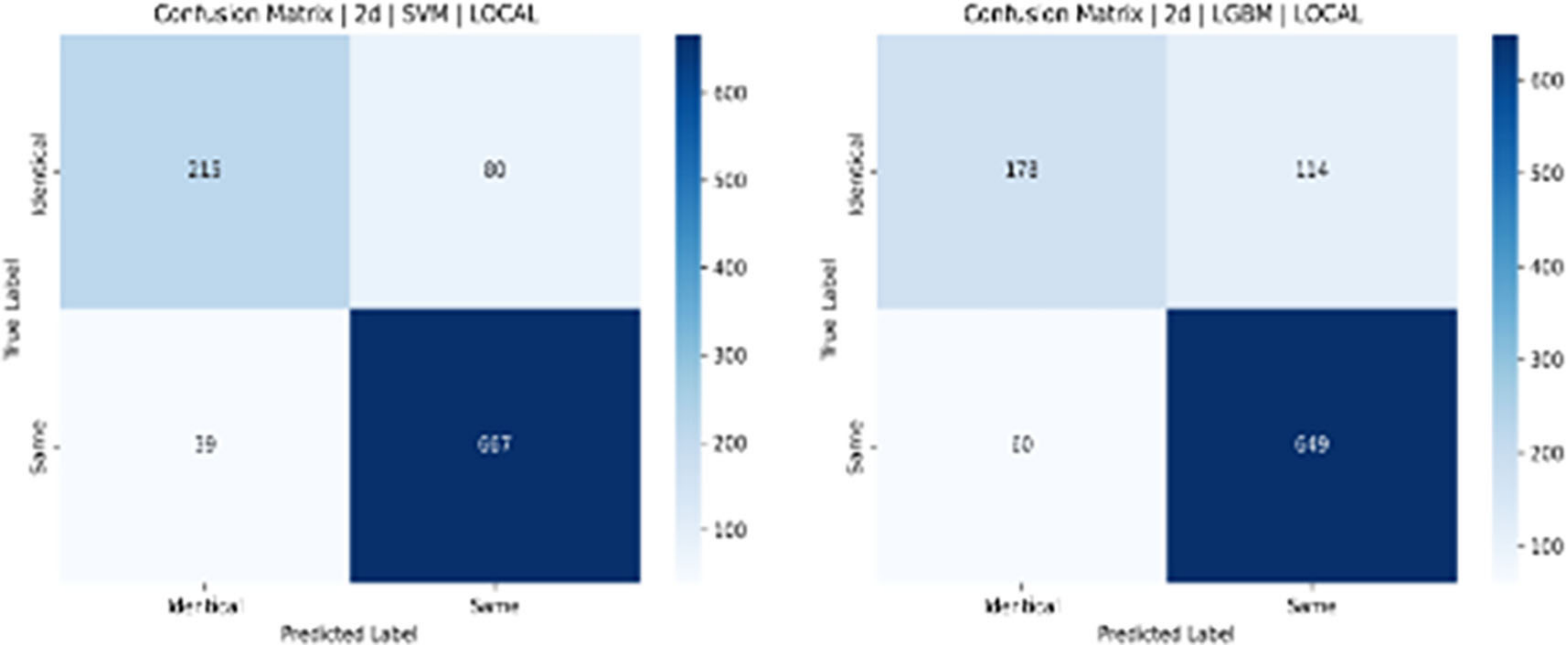
[A] Confusion matrix for SIFT, SURF, and ORB combination with SVM on the ND TWINS dataset; [B] Confusion matrix for SIFT, SURF, and ORB combination with LGBM on the ND TWINS dataset.

**Figure 18.**
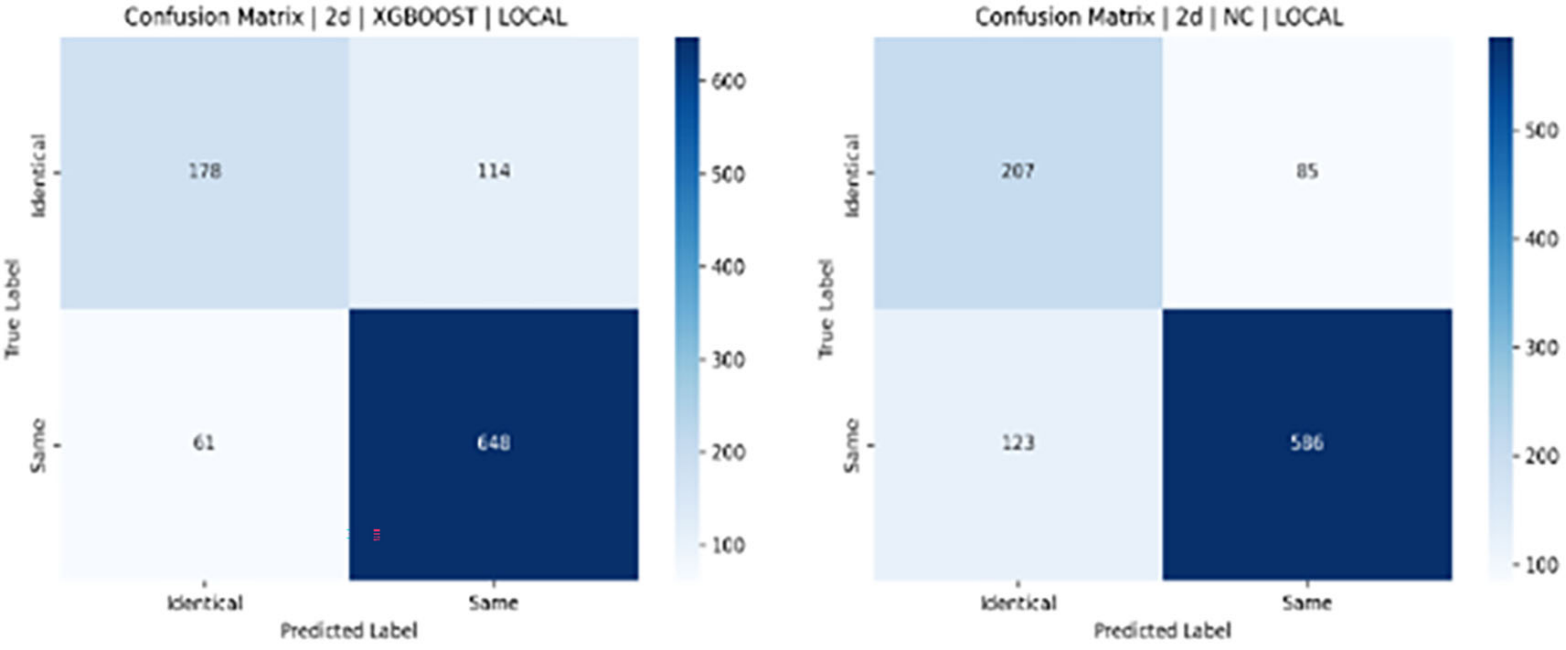
[A] Confusion matrix for SIFT, SURF, and ORB combination with XGBoost on the ND TWINS dataset; [B] Confusion matrix for SIFT, SURF, and ORB combination with NC on the ND TWINS dataset.

1. **Experimental results: Facial images from the ND TWINS dataset.** Table 4 compares the accuracy with which identical twins’/same face photographs can be identified using a simple combination of numerous descriptors such as SIFT, SURF, ORB, SIFT+SURF, SIFT+ORB, SURF+ORB, and SIFT+SURF+ORB. Figures 17 and 18 illustrate the confusion matrices for identical twins’/same facial images when paired with SIFT, SURF, and ORB.

**Table 4.** Effectiveness of SIFT, SURF, ORB, and their combinations on the ND TWINS dataset.

Sl No	Descriptors/ML Models	SVC	LGBM	XGBoost	NC
1	SIFT	78%	75%	75%	72%
2	SURF	76%	73%	74%	71%
3	ORB	80%	79%	77%	76%
4	SIFT+SURF	83%	79%	81%	76%
5	SURF+ORB	84%	81%	80%	78%
6	ORB+SIFT	85%	82%	81%	80%
7	SIFT+SURF+ORB	88%	83%	83%	79%

**Figure 19.**
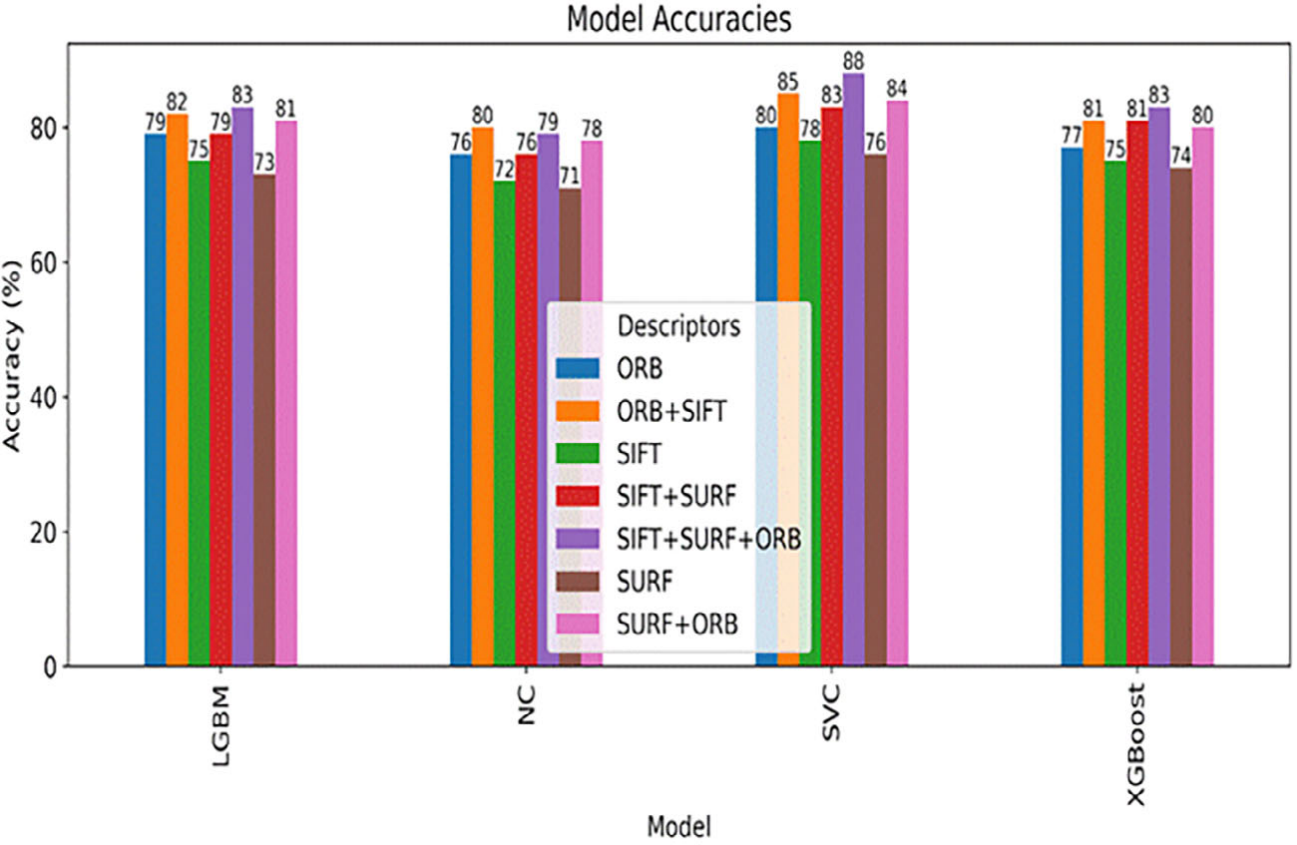
Accuracy comparison bar chart of classifiers using SIFT, SURF, ORB, and their combinations on the ND TWINS dataset.

**Figure 20.**
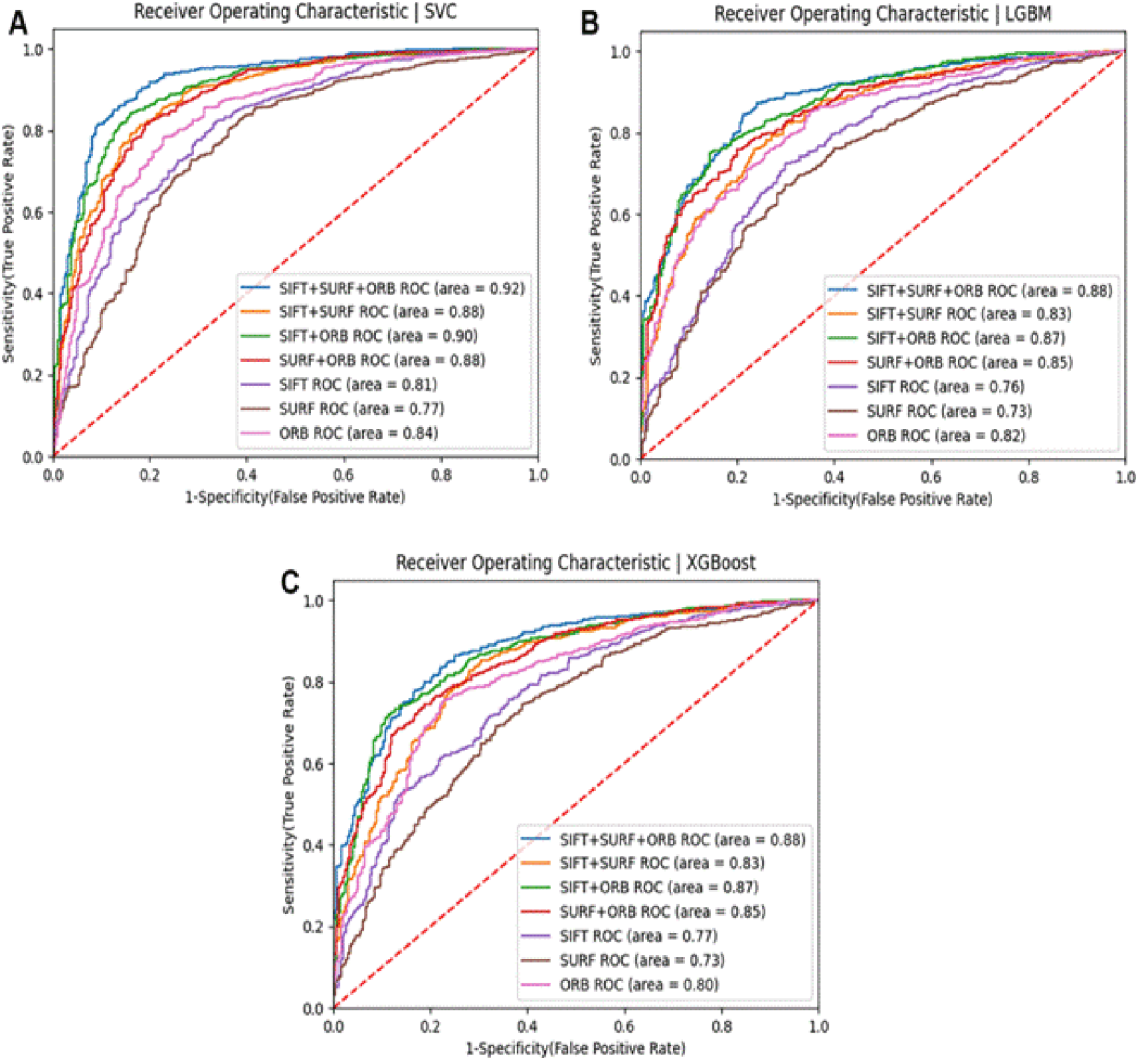
[A] ROC curve of SVM classifiers using SIFT, SURF, and ORB descriptors on the ND TWINS dataset; [B] ROC curve of LGBM classifiers using SIFT, SURF, and ORB descriptors on the ND TWINS dataset; [C] ROC curve of XGBoost classifiers using SIFT, SURF, and ORB descriptors on the ND TWINS dataset.

Figure 19 shows a bar chart comparing the accuracy of various classifiers, such as SVM, LGBM, XGBoost, and NC Classifiers, for identical twins’/same facial images, taking into account SIFT, SURF, ORB, and their combinations on the ND TWINS dataset. Figures 20[A], 20[B], and 20[C] show the ROC curves of classifiers such as SVM, LGBM, and XGBoost for identical twins/the same categories of face pictures using SIFT, SURF, ORB, and combinations in the ND TWINS dataset.

The experiment found that a hybrid feature extraction method combining SIFT, SURF, and ORB can achieve a high recognition accuracy of 88% when combined with a Support Vector Machine, the most accurate classification model to predict identical twin images. This accuracy can be further increased by taking into account the optimization of hyperparameters and considering a larger sample size of images in the dataset.

2. **Experimental results: Facial images from the 3D TEC dataset.** The results of face recognition using the SIFT, SURF, and ORB feature descriptors, as well as combinations of these feature descriptors using the SVM model, are displayed below. Table 5 demonstrates the accuracy of classifying identical twins or matching facial images by combining multiple descriptors with the SVM classifier, using the 3D TEC dataset.

**Table 5.** Effectiveness of SIFT, SURF, ORB, and their combinations on the 3D TEC dataset.

Sl No	Descriptors/ML Models	SVC	LGBM	XGBoost	NC
1	SIFT	66%	65%	64%	62%
2	SURF	64%	62%	63%	61%
3	ORB	67%	66%	65%	66%
4	SIFT+SURF	68%	66%	78%	66%
5	SURF+ORB	69%	70%	69%	69%
6	ORB+SIFT	70%	71%	70%	71%
7	SIFT+SURF+ORB	74%	72%	72%	70%

**Figure 21.**
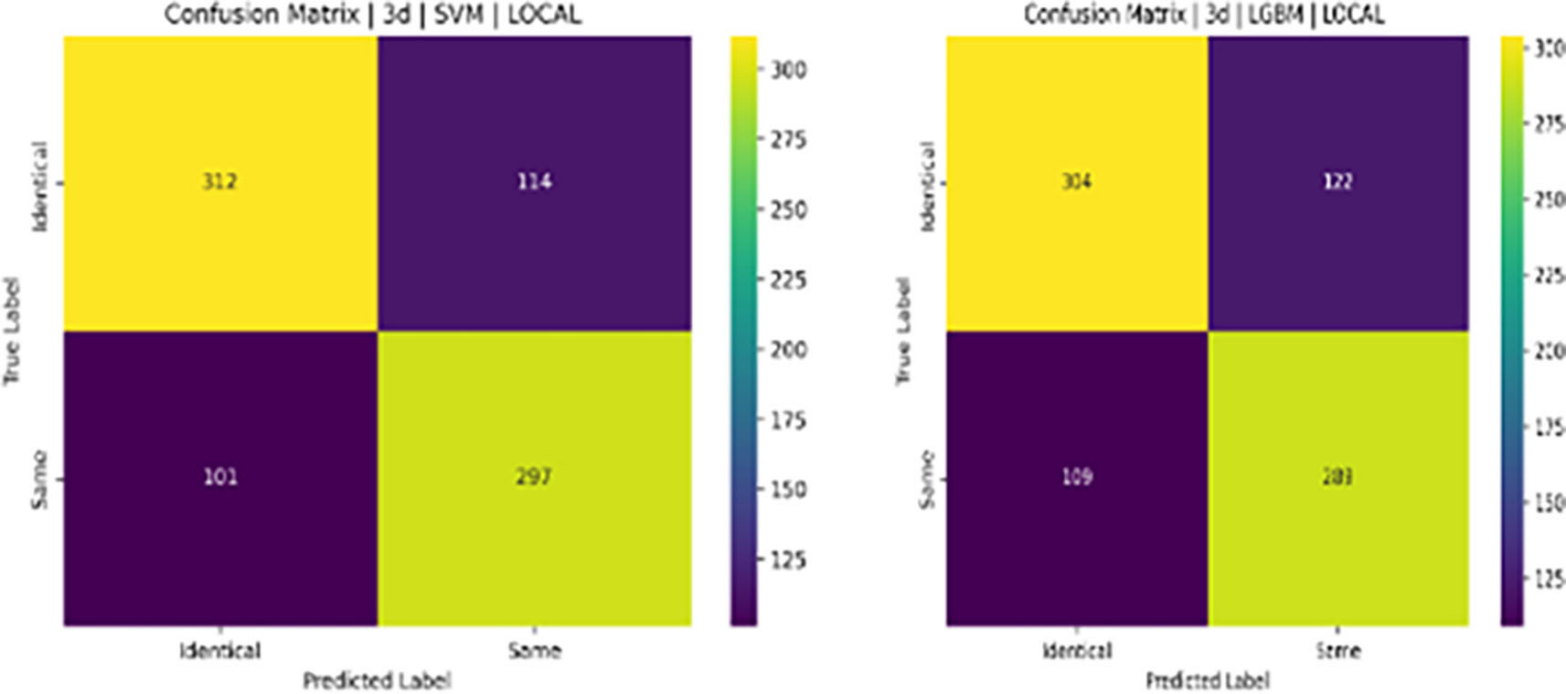
[A] Confusion matrix for SIFT, SURF, and ORB with SVM on the 3D TEC dataset; [B] Confusion matrix for SIFT, SURF, and ORB with LGBM on the 3D TEC dataset.

**Figure 22.**
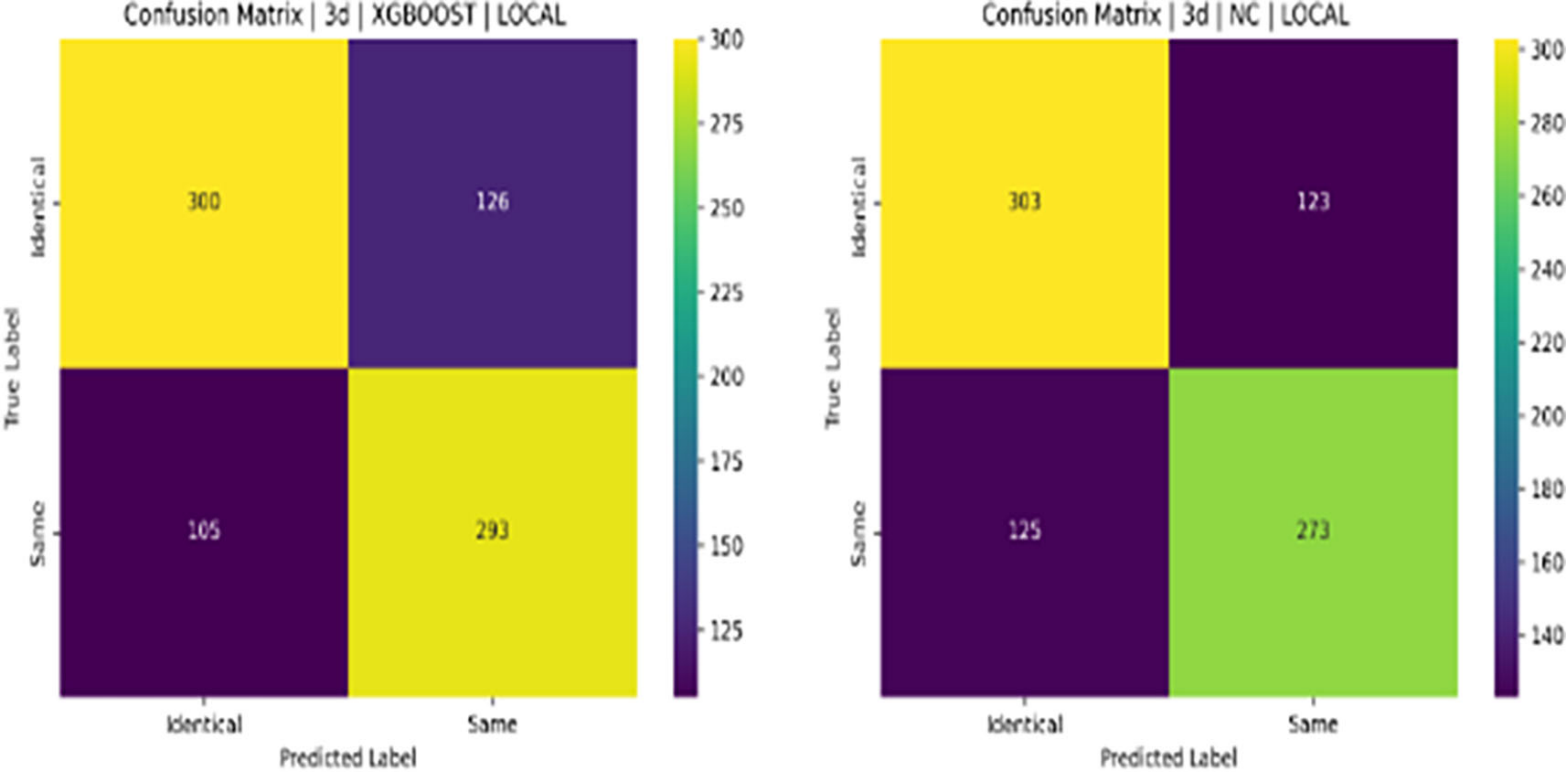
[A] Confusion matrix for SIFT, SURF, and ORB with XGBoost on the 3D TEC dataset; [B] Confusion matrix for SIFT, SURF, and ORB with NC on the 3D TEC dataset.

**Figure 23.**
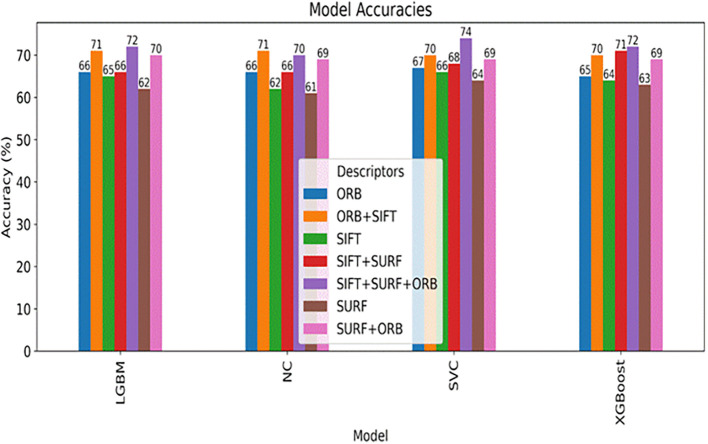
Accuracy comparison bar chart of classifiers using SIFT, SURF, ORB, and their combinations on the 3D TEC dataset.

**Figure 24.**
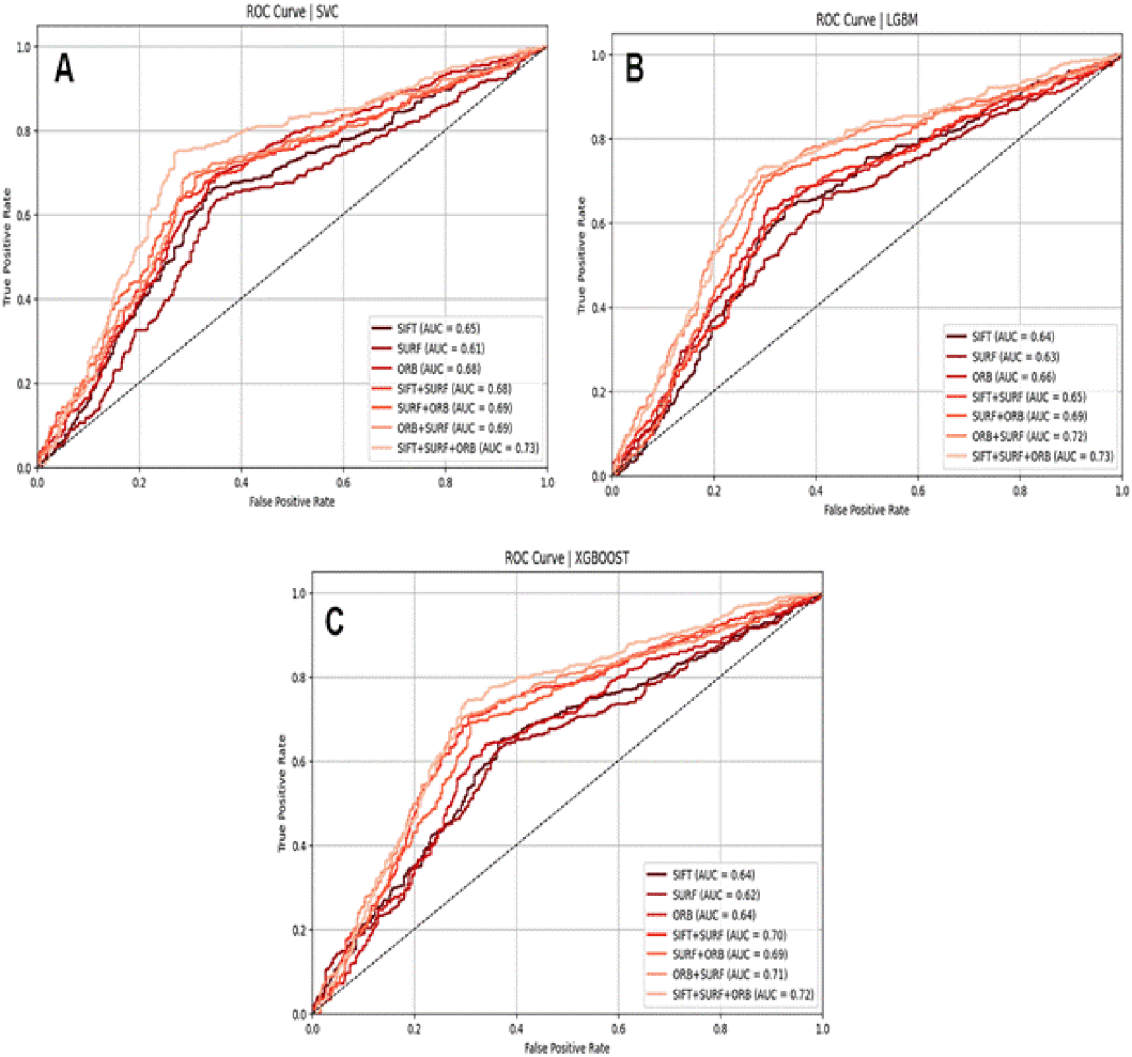
[A] ROC curve of SVM classifiers using SIFT, SURF, and ORB descriptors on the 3D TEC dataset; [B] ROC curve of LGBM classifiers using SIFT, SURF, and ORB descriptors on the 3D TEC dataset; [C] ROC curve of XGBoost classifiers using SIFT, SURF, and ORB descriptors on the 3D TEC dataset.

The confusion matrices for the facial images of identical twins/the same for the combination of SIFT, SURF, and ORB considering SVM and LGBM are shown in Figure 21. The confusion matrices for identical twins’/same’ facial images for the combinations of SIFT, SURF, and ORB, considering the XGBoost and NC classifier, are shown in Figure 22. Figure 23 shows a bar chart of various classifiers, such as SVM, LGBM, XGBoost, and NC Classifiers, for identical twins/same facial images, taking into account SIFT, SURF, ORB, and their combinations on the 3D TEC dataset. Figures 24[A], 24[B], and 24[C] show the ROC curves of classifiers such as SVM, LGBM, and XGBoost for identical twins/same categories of face pictures from the 3D TEC dataset utilizing SIFT, SURF, ORB, and combinations.

The results of the experiment also indicate that a hybrid feature extraction method that incorporates the advantages of SIFT, SURF, and ORB can achieve a high recognition accuracy of 74% when used with a Support Vector Machine. This accuracy can be improved further by optimizing hyperparameters and taking into consideration a greater sample size of pictures in the dataset. This study introduces a novel approach by integrating well-known methods such as SIFT, SURF, ORB, and SVM for the identification of monozygotic twins, a task made challenging by their high visual similarity. A region-wise landmark-based technique is used to extract significant facial proportions, enhancing discriminatory power compared to previous studies. In addition, various classifiers, including LGBM, NC, and XGBoost, are examined, and a hybrid feature extraction strategy is used to improve recognition performance.

This research provides a comprehensive comparative analysis of multiple methods for this specific application offering valuable insights that have not yet been explored in this domain.

## 7. Conclusion and future work

The research uses SIFT, SURF, and ORB feature descriptors and different combinations of these descriptors to examine identical twin face recognition results. The ND Twins data set and 3D datasets were used for the studies, and the SVM, LGBM, XGBoost, and NC classifiers were used. Area Under the Curve (AUC), True Positive Rate (TPR), False Positive Rate (FPR), and recognition accuracy were the four metrics used to evaluate the effectiveness of these approaches. The findings show that ORB is superior to SIFT and SURF in identical twin recognition, even though it extracts fewer characteristics. Furthermore, combining SIFT, SURF, and ORB data with a Support Vector Machine (SVM) classifier produces better results than other cutting-edge techniques for identical twin recognition. Future work will concentrate on creating a multimodal facial recognition system that uses several modalities to solve the problem of identifying faces that are like each other, including identical twins and lookalikes. In the future, a larger dataset of face photos with the best classifier will be created, allowing researchers to look at more precise methods to raise the standard of the current study. Future work will also focus on evaluating the method across multiple datasets to assess its generalizability and effectiveness in different real-world scenarios.

## Author contributions

GS: authoring - Original draft preparation (creation, preparation, and presentation of the published work, mainly writing the first draft); KP: Writing - Writing critique and editing; SRP: Supervision; VN: Supervision, Funding; AJ: editing; funding. All authors have read and agreed to the published version of the manuscript.

## Ethical approval statement

This study did not involve the collection of data from human participants, requiring ethical approval. The research was conducted using publicly available datasets, including the ND Twins and 3D TEC datasets. As no direct human subject research was conducted, ethical approval was not required. However, this study adheres to the ethical principles outlined in the Declaration of Helsinki. We have properly acknowledged all sources of the datasets and images and have used them by their respective terms of use and data-sharing policies. ^60^ ND Twins and 3D TEC datasets have been accessed based on the University of Notre Dame Biometrics Database Release Agreement signed between Notre Dame University and Manipal Academy of Higher Education dated 21/2/2023. A copy of the agreement is submitted to the Editorial Team of the Journal.

Figure permissions: The ND Twins and 3D TEC datasets have been accessed based on the University of Notre Dame Biometrics Database Release Agreement signed between Notre Dame University and Manipal Academy of Higher Education dated 21/2/2023. A copy of the agreement is submitted to the Editorial Team of the Journal.

## Data Availability

The datasets ND-Twins-2009-2010 and 3D TEC datasets require a license agreement signed by the university to gain access to the dataset. The link for the same is as follows:
1. ND-Twins-2009-2010: Available [online]: https://cvrl.nd.edu/projects/data/#nd-twins-2009-2010
2. 3D TEC: Available [online]: https://cvrl.nd.edu/projects/data/#3d-twins-expression-challenge-3d-tec-data-set The ND-Twins-2009-2010 and 3D TEC datasets used in this study were obtained from the University of Notre Dame. However, access to these datasets is restricted as they were utilized under a licensing agreement and are not publicly accessible. However, the data may be available upon a reasonable request, subject to approval from the University of Notre Dame and the authors. Access to these datasets is granted under the following conditions:
1. The requesting researcher must be affiliated with an accredited academic or research institution. 2. The intended use must be for non-commercial academic research purposes only. 3. The researcher’s institution must sign a formal license agreement that includes: 4. A commitment to maintain data confidentiality 5. Agreement not to redistribute the data to third parties 6. Commitment to cite the original dataset in any resulting publications 7. Agreement to use the data only for the specified research purpose 8. The researcher must provide a brief description of the research project for which the data will be used. 9. All project team members who will access the data must be listed in the application. The license agreement approval process typically takes 2-4 weeks. Once approved, access credentials are provided to download the datasets from the secure University of Notre Dame servers. Access to these datasets was obtained through a formal license agreement between the research institution and the University of Notre Dame. Data transfer was carried out securely via the Globus research data transfer platform ( https://www.globus.org/), which ensures secure and authenticated access to research data sets. The research team strictly adhered to all conditions specified in the license agreement, including data protection measures and redistribution restrictions.
