# Supplementary material for: Region-wise landmarks-based feature extraction employing SIFT, SURF, and ORB feature descriptors to recognize Monozygotic twins from 2D/3D Facial Images
Source: F1000Res. 2025 Jun 20;14:444. Originally published 2025 Apr 16. [Version 2] doi: 10.12688/f1000research.162911.2 (PMC12334920; doi:10.12688/f1000research.162911.2)
Supplement: Peer review history

# Reviewer report: Reviewer response for version 2

**DOI:** 10.5256/f1000research.182977.r398327

**Published:** 2025-08-12

**Authors:** Siew Chin Chong

**Affiliations:** 1 Multimedia University, Bukit Beruang, Malaysia

This paper presents a face recognition approach for monozygotic twins using region-specific facial landmarks and three feature descriptors (SIFT, SURF, ORB), with classification performed by SVM, XGBoost, LGBM, and Nearest Centroid. 

 I would like to commend the authors for presenting a comprehensive and well-structured manuscript that reads almost like a detailed lecture note. The systematic breakdown of the methodology—from facial landmark extraction using MediaPipe to the application of classical feature descriptors (SIFT, SURF, ORB) and subsequent classification techniques—demonstrates clarity and pedagogical value. Each component is introduced with sufficient background and justification, making the paper accessible not only to seasoned researchers but also to readers new to the domain of facial recognition.

 While the manuscript presents a well-structured and detailed methodology, I suggest that the authors include a more explicit comparison between the proposed approach and existing benchmark methods which have been discussed in the literature review section. The comparison would provide readers with a clearer perspective on where the proposed system stands within the current landscape of monozygotic twin face recognition research.

Is the work clearly and accurately presented and does it cite the current literature?

Yes

If applicable, is the statistical analysis and its interpretation appropriate?

Yes

Are all the source data underlying the results available to ensure full reproducibility?

Yes

Is the study design appropriate and is the work technically sound?

Yes

Are the conclusions drawn adequately supported by the results?

Yes

Are sufficient details of methods and analysis provided to allow replication by others?

Yes

Reviewer Expertise:

-

I confirm that I have read this submission and believe that I have an appropriate level of expertise to confirm that it is of an acceptable scientific standard.

# Reviewer report: Reviewer response for version 2

**DOI:** 10.5256/f1000research.182977.r398326

**Published:** 2025-08-08

**Authors:** Muhammad Arslan Rauf

**Affiliations:** 1 University of Electronic Science and Technology of China, Chengdu, Sichuan, China

1. The title should be concise yet informative. If it's too lengthy or vague, consider summarizing it to maintain reader interest and clarity.

 2. Table 4 is titled "Effectiveness of SIFT, SURF, ORB, and their combinations on the ND TWINS dataset," but it lacks a clear, descriptive caption that specifies it reports accuracy or other performance metrics.

 3. Table 5 is titled "Effectiveness of SIFT, SURF, ORB, and their combinations on the 3D TEC dataset," yet again, it does not explicitly mention that the accuracy values are the metric being presented.

 4. The visualization in Figure 16 is not sufficiently clear and requires improved labeling and presentation to better illustrate the differences in features.

 5. The observations about headings 4 and 5 suggest a need for clearer differentiation or restructuring; integrating methodology into section 4 and experimental subsections into section 5 would improve the organization and clarity of the document.

 6.The document mentions the dataset sources but does not provide a link to the code or implementation details for the feature extraction and classification pipelines. To enhance reproducibility and validate the results, the authors are encouraged to share their code or detailed implementation parameters either as supplementary material or via a publicly accessible repository. This would facilitate peer validation and further research based on their work.

 7. To include the latest citations, the authors should review recent publications in facial recognition, dataset licensing, and biometric analysis from 2024 and 2025 and update the reference list accordingly.

 8. After these minor revisions, the paper will be acceptable.

Is the work clearly and accurately presented and does it cite the current literature?

Partly

If applicable, is the statistical analysis and its interpretation appropriate?

Yes

Are all the source data underlying the results available to ensure full reproducibility?

Partly

Is the study design appropriate and is the work technically sound?

Yes

Are the conclusions drawn adequately supported by the results?

Yes

Are sufficient details of methods and analysis provided to allow replication by others?

Partly

Reviewer Expertise:

Deep Learning, Computer Vision, Recommender Systems

I confirm that I have read this submission and believe that I have an appropriate level of expertise to confirm that it is of an acceptable scientific standard.

# Reviewer report: Reviewer response for version 2

**DOI:** 10.5256/f1000research.182977.r393393

**Published:** 2025-06-23

**Authors:** Dhimas Tribuana

**Affiliations:** 1 Universitas Komputer Indonesia, Bandung, Indonesia

**1. Importance of the Manuscript for the Scientific Community**

 This manuscript contributes significantly to the field of biometric and forensic identification by addressing the complex challenge of recognizing monozygotic twins using facial features. The use of region-wise landmark extraction combined with handcrafted local descriptors (SIFT, SURF, ORB) offers an interpretable and efficient approach with high practical relevance. The work presents a well-defined methodology with sound experimental validation, making it valuable to researchers and practitioners alike.

**2. Title Suitability**

 Yes, the title clearly and accurately reflects the core objective and methodology of the study.

**3. Abstract Evaluation**

 The abstract is clear, informative, and well-balanced. It successfully summarizes the objectives, datasets, methods, classifiers, and the primary findings. The inclusion of quantitative results (e.g., 88% accuracy with SVM) enhances clarity and relevance.

**4. Scientific Correctness**

 The study is methodologically sound. The authors provide comprehensive details on feature extraction, preprocessing, landmark selection, and classifier configurations. The experimental design, including dataset characteristics and evaluation metrics, is appropriate and clearly articulated.

**5. References Sufficiency and Recency**

 The literature review has been significantly improved in the revised manuscript. It now includes several recent and relevant studies published between 2022 and 2024, addressing both traditional and deep learning approaches. The references are appropriate and sufficient.

**6. Language Quality**

 The manuscript is written in clear and precise academic English. The language is appropriate for publication, with only minor improvements suggested for clarity and flow.

**7. Optional/General Comments**

- The addition of statistical hypothesis testing (e.g., t-test, Mann-Whitney U test) strengthens the empirical analysis.
- The methodological transparency—particularly the explanation of feature vector construction and classifier tuning—is commendable.
- Overall, the manuscript is well-organized and presents a cohesive narrative from problem definition to conclusion.

**Part 2: Ethical and Competing Interest Issues**

**1. Ethical Issues**

 No ethical concerns are identified. The study uses publicly available datasets, and all data are anonymized appropriately.

**2. Competing Interests**

 There are no declared competing interests. None are apparent.

**3. Plagiarism**

 The manuscript is original, and all sources are properly cited. No issues of plagiarism are identified.

**Part 3: Declaration of Competing Interest of the Reviewer**

 I declare that I have no competing interest related to this manuscript.

**Part 4: Objective Evaluation**

 Criterion Evaluation Is the work clearly and accurately presented, and does it cite current literature? ✅ Yes

 Is the study design appropriate and technically sound?✅ Yes

 Are sufficient methodological details provided to allow replication? ✅ Yes

 If applicable, is the statistical analysis and its interpretation appropriate? ✅ Yes

 Are all the source data underlying the results available? ✅ Yes

 Are the conclusions adequately supported by the results? ✅ Yes

Is the work clearly and accurately presented and does it cite the current literature?

Partly

If applicable, is the statistical analysis and its interpretation appropriate?

Partly

Are all the source data underlying the results available to ensure full reproducibility?

No

Is the study design appropriate and is the work technically sound?

Yes

Are the conclusions drawn adequately supported by the results?

Yes

Are sufficient details of methods and analysis provided to allow replication by others?

Partly

Reviewer Expertise:

Computer Vision; Biometrics; Machine Learning; Pattern Recognition

I confirm that I have read this submission and believe that I have an appropriate level of expertise to confirm that it is of an acceptable scientific standard.

# Reviewer report: Reviewer response for version 1

**DOI:** 10.5256/f1000research.179184.r382141

**Published:** 2025-05-19

**Authors:** Dhimas Tribuana

**Affiliations:** 1 Universitas Komputer Indonesia, Bandung, Indonesia

**Summary:**

 This study investigates the use of region-wise facial landmark features, extracted using MediaPipe and described using SIFT, SURF, and ORB, for the recognition of monozygotic twins. The proposed method is evaluated using both 2D and 3D facial image datasets (ND Twins and 3D TEC, respectively) and classified with four different machine learning models (SVM, XGBoost, LGBM, and Nearest Centroid). The results indicate that the highest accuracy (88%) is achieved using 2D features and SVM.

**Recommendations for Revision:**

1. **Literature Review:** The authors should include more recent and relevant studies, especially those that explore deep learning approaches or similar hybrid methods.
2. **Methodological Details:** Provide specific technical details (e.g., parameters, feature vector dimensions, classifier settings, data splitting strategy, preprocessing steps) to support replication.
3. **Statistical Analysis:** Incorporate statistical measures such as standard deviation or confidence intervals and consider performing tests for statistical significance.
4. **Reproducibility:** Make the source code and datasets (or dataset access links) publicly available in a supplementary repository.

Is the work clearly and accurately presented and does it cite the current literature?

Partly

If applicable, is the statistical analysis and its interpretation appropriate?

Partly

Are all the source data underlying the results available to ensure full reproducibility?

No

Is the study design appropriate and is the work technically sound?

Yes

Are the conclusions drawn adequately supported by the results?

Yes

Are sufficient details of methods and analysis provided to allow replication by others?

Partly

Reviewer Expertise:

Computer Vision; Biometrics; Machine Learning; Pattern Recognition

I confirm that I have read this submission and believe that I have an appropriate level of expertise to confirm that it is of an acceptable scientific standard, however I have significant reservations, as outlined above.

# Response

**Published:** 2025-05-28

**Authors:** Vinod Nayak

**Affiliations:** ForensicMedicine andToxicology, Manipal Academy of Higher Education, Manipal, Karnataka, India

**Dhimas Tribuana**, Universitas Komputer Indonesia, Bandung, Indonesia 

**Summary:**

 This study investigates the use of region-wise facial landmark features, extracted using MediaPipe and described using SIFT, SURF, and ORB, for the recognition of monozygotic twins. The proposed method is evaluated using both 2D and 3D facial image datasets (ND Twins and 3D TEC, respectively) and classified with four different machine learning models (SVM, XGBoost, LGBM, and Nearest Centroid). The results indicate that the highest accuracy (88%) is achieved using 2D features and SVM.

**Recommendations for Revision:**

1. **Literature Review:** The authors should include more recent and relevant studies, especially those that explore deep learning approaches or similar hybrid methods.

**The following papers are included in the manuscript**

 1 Nahar, B. Abul-Huda, Bataineh A. Al., and Al-Khatib R. M., “Twins and Similar Faces Recognition Using Geometric and Photometric Features with Transfer Learning,” International Journal of Computing and Digital Systems, vol. 11, no. 1, pp. 129–139, Jan. 2022, doi: 10.12785/ijcds/110110.

 2 Vengatesan K, Kumar A, Karuppuchamy V, Shaktivel R, and Singhal A, "Face Recognition of Identical Twins Based on Support Vector Machine Classifier," 2019 Third International Conference on I-SMAC (IoT in Social, Mobile, Analytics and Cloud) (I-SMAC), 2019, pp. 577-580, doi: 10.1109/I-SMAC47947.2019.9032548.

 3 Parde C. J. et al., “Twin Identification over Viewpoint Change: A Deep Convolutional Neural Network Surpasses Humans,” ACM Transactions on Applied Perception, vol. 20, no. 3, pp. 1–15, Jul. 2023, doi: 10.1145/3609224

 4 Tribuana, Dhimas, Hazriani Hazriani, and Abdul Latief Arda. "Face recognition for smart door security access with convolutional neural network method." *TELKOMNIKA (Telecommunication Computing Electronics and Control)* 22.3 (2024): 702-710.

 5 Ozbilen, E. Basal, Yilmaz, H. N., and S. Biren, “Facial morphology differences in monozygotic twins: a retrospective stereophotogrammetric study,” The Angle Orthodontist, vol. 93, no. 6, pp. 706–711, Jul. 2023, doi: 10.2319/120722-840.1.

  

1. **Methodological Details:** Provide specific technical details (e.g., parameters, feature vector dimensions, classifier settings, data splitting strategy, preprocessing steps) to support replication.

These Methodological Details are included wherever required **.**

**1. Landmark Detection with MediaPipe:**

- **Tool**: MediaPipe Face Mesh.
- **Version**: MediaPipe v0.8.11
- **Landmark Points**: 468 2D facial landmarks extracted per image. (full face mesh)
- **Region selection strategy**: Define specific landmark indices for facial regions. Only relevant landmark subsets were retained (e.g., eyes, nose, mouth).
  - **Eye regions:** Landmarks 130, 27, 243, 23 (right eye), 463, 257, 359, 253 (left eye)
  - **Eyebrow regions** (left eyebrow) 276, 283, 282, 295, 285, 300, 293, 334, 296, 336, (right eyebrow ) 46, 53, 52,  65, 55, 70, 63, 105, 66, 107.
  - **Nose region:** Landmarks 8, 240, 98, 164, 327, 460, 8
  - **Mouth region**: Landmarks 61, 146, 46, 91, 181, 84, 17, 314, 405, 321, 375, 291, 65, 185, 40, 39, 37, 0, 267, 269, 270, 409, 291, 78, 95, 88, 178, 87, 14, 317, 402, 318, 324, 308, 78, 191, 80, 81, 82, 13, 312, 311, 310, 415, 308
  - **Jawline:** Landmarks 138, 214, 212, 57, 61, 14, 291, 287, 432, 434, 367, 379,400, 377, 152, 148, 176, 149, 138
  - **Forehead:** Landmarks 10, 151, 9, 107-109
- **Resolution**: All images were resized to a consistent resolution (e.g., 256×256) before landmark extraction.

**2. Key point Matching using SIFT, SURF, and ORB:**

- **Descriptors Used:** OpenCV Version: 4.6.0
  - SIFT (Scale-Invariant Feature Transform)
  - SURF (Speeded-Up Robust Features)
  - ORB (Oriented FAST and Rotated BRIEF)
- **Parameters:**

- SIFT: nfeatures=100/500, contrastThreshold=0.04, edgeThreshold: 10, sigma: 1.6
- SURF: hessianThreshold=400, nOctaves: 4, nOctaveLayers: 3, extended: False, upright: False
- ORB: nfeatures=100/1000, scaleFactor=1.2, nlevels=8, edgeThreshold: 31, firstLevel: 0, WTA_K: 2, patchSize: 31.

- **Matching Strategy:** Brute-force matching
- **Match Quality:** Ratio test (Lowe’s ratio test) with a threshold of 0.75 for good matches.
- **Distance Threshold:** Maximum distance = 50 pixels for matched points
- **Output:** Number of matched and unmatched points per method.
- **Feature Construction:**
  - **Final Feature Vector Dimension:** 16 × 3 = 48 features. The 16 features were derived from geometric and statistical analysis on matched/mismatched points.
  - Each feature was represented with three attributes (e.g., mean distance, standard deviation, count).

**3. Datasets Used:**

 2D Dataset: **ND Twins Dataset**

- **Total samples**: 24000 images
- **Image specifications**:
  - Resolution: [e.g., 1024×768 pixels]
  - Format: JPEG/PNG

3D Dataset: **3D TEC Dataset**

- Total samples: 107 pairs
- 3D data specifications:
  - Format: Point clouds/3D meshes
  - Resolution: 512×512 pixels
  - Depth map conversion to grayscale images

**4. Data Splitting Strategy**

- **Train-Test Split:** 80:20 ratio.

**5. Classifiers and Parameters:**

**Support Vector Machine (SVM)**

- Kernel: Radial Basis Function (RBF), C = 1.0, gamma = ‘scale’
- Hyperparameters:
  - C: 10.0 (grid search range: [0.1, 1, 10, 100, 1000])
  - gamma: 0.01 (grid search range: [0.001, 0.01, 0.1, 1, 'scale'])
  - class_weight: 'balanced'
- Implementation: scikit-learn 1.1.0

**XGBoost**

 max_depth=6, learning_rate=0.1, n_estimators=100

- Hyperparameters:
  - n_estimators: 200
  - max_depth: 6
  - learning_rate: 0.1
  - subsample: 0.8
  - colsample_bytree: 0.8
  - min_child_weight: 1
  - reg_alpha: 0.1
  - reg_lambda: 1.0
  - random_state: 42
- Implementation: XGBoost 1.6.2

**LightGBM**

 num_leaves=31, learning_rate=0.1, n_estimators=100

- Hyperparameters:
  - num_leaves: 31
  - learning_rate: 0.05
  - feature_fraction: 0.9
  - bagging_fraction: 0.8
  - bagging_freq: 5
  - min_data_in_leaf: 10
  - lambda_l1: 0.1
  - lambda_l2: 0.1
  - random_state: 42
- Implementation: LightGBM 3.3.3

**Nearest Centroid**

- Metric: Euclidean distance
- No hyperparameters tuned.
- Implementation: scikit-learn 1.1.0

 

1. **Statistical Analysis:** Incorporate statistical measures such as standard deviation or confidence intervals and consider performing tests for statistical significance.

To explore and validate the statistical significance of differences between the two classes (same person vs identical twin), a comprehensive statistical analysis was conducted on all features in the dataset used to classify whether two facial images represent the same person (label = 0) or identical twins (label = 1).

**Feature**

**Mean (0)**

**Mean (1)**

**T-test p-value**

**Mann-Whitney p-value**

 ORB_NOSE_MIS_MATCH

 0.0352

 0.0720

 1.21e-71

 2.25e-93

 ORB_L_EYE_MIS_MATCH

 0.0352

 0.0666

 1.48e-59

 2.99e-72

 ORB_R_EYE_MIS_MATCH

 0.0366

 0.0678

 3.84e-57

 1.40e-66

 SIFT_OVAL_FACE_MIS_MATCH

 0.0661

 0.1126

 1.05e-51

 8.47e-53

 SURF_OVAL_FACE_MIS_MATCH

 0.0361

 0.0583

 9.32e-45

 1.23e-41

 SURF_FACE_CURVE_MIS_MATCH

 0.0312

 0.0490

 1.30e-40

 4.99e-39

 ORB_FACE_CURVE_MIS_MATCH

 0.0455

 0.0748

 4.92e-38

 2.06e-43

 ORB_L_EYE_MATCH

 0.1168

 0.0861

 5.11e-34

 8.10e-23

 SIFT_R_EYE_MIS_MATCH

 0.0367

 0.0553

 5.15e-33

 1.39e-35

 ORB_LIPS_MIS_MATCH

 0.0362

 0.0564

 1.04e-32

 6.30e-40

 To explore and validate the statistical significance of differences between the two classes (same person vs identical twin), a comprehensive statistical analysis was conducted on all features in the dataset used to classify whether two facial images represent the same person (label = 0) or identical twins (label = 1).

**Descriptive Statistics**: For each feature, descriptive statistics were computed separately for each class as shown in table 3. These included Mean and Standard Deviation to describe central tendency and variability. Also 95% Confidence Intervals (CI) for the mean, calculated using t-distribution-based methods, to estimate the range within which the true population means is expected to lie with 95% certainty.

 Table 3. Top 10 most statistically significant features that differ between same person (0) and identical twin (1) classes:

**Feature**

**Mean (0)**

**Mean (1)**

**T-test p-value**

**Mann-Whitney p-value**

 ORB_NOSE_MIS_MATCH

 0.0352

 0.0720

 1.21e-71

 2.25e-93

 ORB_L_EYE_MIS_MATCH

 0.0352

 0.0666

 1.48e-59

 2.99e-72

 ORB_R_EYE_MIS_MATCH

 0.0366

 0.0678

 3.84e-57

 1.40e-66

 SIFT_OVAL_FACE_MIS_MATCH

 0.0661

 0.1126

 1.05e-51

 8.47e-53

 SURF_OVAL_FACE_MIS_MATCH

 0.0361

 0.0583

 9.32e-45

 1.23e-41

 SURF_FACE_CURVE_MIS_MATCH

 0.0312

 0.0490

 1.30e-40

 4.99e-39

 ORB_FACE_CURVE_MIS_MATCH

 0.0455

 0.0748

 4.92e-38

 2.06e-43

 ORB_L_EYE_MATCH

 0.1168

 0.0861

 5.11e-34

 8.10e-23

 SIFT_R_EYE_MIS_MATCH

 0.0367

 0.0553

 5.15e-33

 1.39e-35

 ORB_LIPS_MIS_MATCH

 0.0362

 0.0564

 1.04e-32

 6.30e-40

**Tests for Statistical Significance: **To rigorously assess whether these differences were statistically significant, two tests were performed for each feature:

1. **Independent Two-Sample t-test: **This test evaluated whether the **mean values** of each feature differ significantly between the two classes. To accommodate unequal variances between groups, Welch’s t-test was used. A p-value < 0.05 was considered statistically significant.
2. **Mann-Whitney U Test**: As a non-parametric alternative, the Mann-Whitney U test was applied to compare the **rank distributions** of the features without assuming normality. This test is particularly robust to non-normal distributions and confirmed the t-test results.

Figure 16. Boxplots for the top 10 statistically significant features

 The statistical tests revealed that several features exhibited **highly significant differences** between the classes, with p-values well below 0.001 in both t-tests and Mann-Whitney tests. The discriminative top 10 features are included. These features had both l **arge differences in mean values**, and **non-overlapping confidence intervals**, indicating strong separation between the two classes. Boxplots were generated for the top features show in Figure 16, visually confirming that features associated with mismatch scores tend to be significantly **higher for identical twins** than for the same person. This validates that the classifier can capture subtle but consistent biometric differences between these groups.

 The incorporation of statistical measures and hypothesis testing provides strong evidence that certain facial features differ significantly between identical twins and the same person. These findings support the reliability of the selected features for classification tasks and justify their inclusion in any downstream machine learning models.

**Reproducibility:** Make the source code and datasets (or dataset access links) publicly available in a supplementary repository.

 Research Methods and implementation procedure is explained in the manuscript in section 5. Also, data access links and procedures are already shared in the data availability part of the manuscript.

  

- **Is the work clearly and accurately presented, and does it cite the current literature?**

Partly

- **Is the study design appropriate and is the work technically sound?**

Yes

- **Are sufficient details of methods and analysis provided to allow replication by others?**

Partly

- **If applicable, is the statistical analysis and its interpretation appropriate?**

Partly

- **Are all the source data underlying the results available to ensure full reproducibility?**

No

- **Are the conclusions drawn adequately supported by the results?**

Yes

 Reviewer Expertise

 Computer Vision; Biometrics; Machine Learning; Pattern Recognition

**We confirm that I have read this submission and believe that we have an appropriate level of expertise to confirm that it is of an acceptable scientific standard.**
